# A Novel Compound, Tanshinol Borneol Ester, Ameliorates Pressure Overload-Induced Cardiac Hypertrophy by Inhibiting Oxidative Stress via the mTOR/β-TrCP/NRF2 Pathway

**DOI:** 10.3389/fphar.2022.830763

**Published:** 2022-02-03

**Authors:** Dongjian Han, Fuhang Wang, Bo Wang, Zhentao Qiao, Xinyue Cui, Yi Zhang, Qingjiao Jiang, Miaomiao Liu, Jiahong Shangguan, Xiaohui Zheng, Yajun Bai, Chunyan Du, Deliang Shen

**Affiliations:** ^1^ Department of Cardiology, The First Affiliated Hospital of Zhengzhou University, Zhengzhou, China; ^2^ Department of Vascular and Endovascular Surgery, The First Affiliated Hospital of Zhengzhou University, Zhengzhou, China; ^3^ Key Laboratory of Resource Biology and Biotechnology in Western China, College of Life Sciences, Northwest University, Xi’an, China; ^4^ Laboratory Animal Center, Academy of Medical Science, Zhengzhou University, Zhengzhou, China

**Keywords:** cardiac hypertrophy, antioxidant, er stress, Nrf2, mTOR, autophagy

## Abstract

Tanshinol borneol ester (DBZ) exerts anti-atherosclerotic and anti-inflammatory effects. However, its effects on cardiac hypertrophy are not well understood. In this work, we investigated the treatment effects and potential mechanisms of DBZ on the hypertrophic heart under oxidative stress and endoplasmic reticulum (ER) stress. A hypertrophic model was established in rats using transverse-aortic constriction (TAC) surgery and in neonatal rat cardiomyocytes (NRCMs) using angiotensin II (Ang II). Our results revealed that DBZ remarkably inhibited oxidative stress and ER stress, blocked autophagy flow, and decreased apoptosis *in vivo* and *in vitro* through nuclear NRF2 accumulation, and enhanced NRF2 stability *via* regulating the mTOR/β-TrcP/NRF2 signal pathway. Thus, DBZ may serve as a promising therapeutic for stress-induced cardiac hypertrophy.

**Graphical Abstract:**
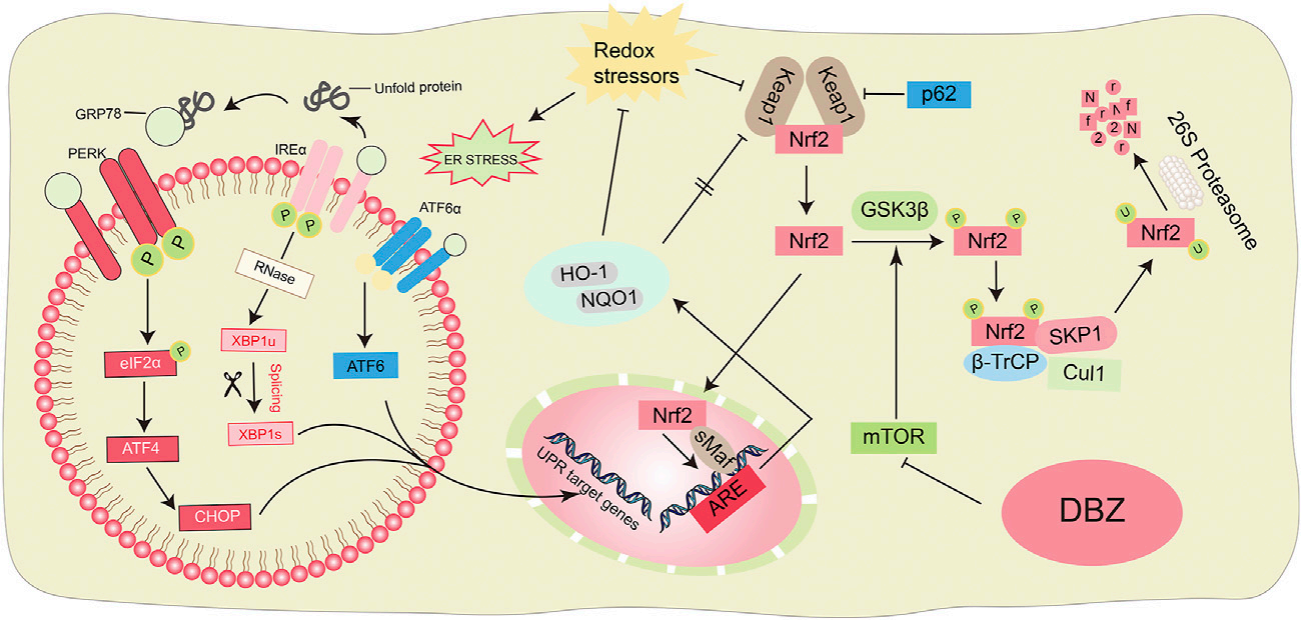
Scheme summarizing the proposed mechanisms for the antioxidant and anti-ER stress of DBZ.

## Introduction

Hypertension, a leading cause of cardiac hypotrophy, is characterized by a compensatory enlargement of cardiomyocytes, eventually followed by the transition to heart failure (HF) (Li et al., 2020). The pathophysiological remodeling of hypertrophy is highly associated with oxidative stress (Raut et al., 2020). Cardiomyocytes require a constant ATP supply to meet their high energy demand and maintain normal cardiac pump function under stress conditions by enhancing mitochondrial oxidative phosphorylation. However, overload of mitochondrial oxidative phosphorylation significantly promotes reactive oxygen species (ROS) generation and intracellular oxidative stress, resulting in mitochondrial dysfunction and even cardiomyocyte apoptosis, eventually leading to HF (Jiang et al., 2021; Walejko et al., 2021).

Endoplasmic reticulum (ER) stress is also an important mechanism responsible for the pathogenesis of cardiac hypertrophy, which is maintained by unfolded protein response (UPR), an adaptive pathway that adjusts intracellular protein-folding capacity to maintain ER homeostasis and cell survival (Cominacini et al., 2015; Xiong et al., 2021). Additionally, extensive studies suggested that perturbation of intracellular oxidative homeostasis can activate ER stress with the imbalance of misfolded and unfolded protein accumulation in the ER lumen, in turn resulting in excessive ROS production and dysfunction of mitochondrial respiration, eventually exacerbating cardiomyocyte apoptosis (Wang et al., 2018; Xia et al., 2021). Although moderate ER stress restores prooxidant-antioxidant balance, maladaptive UPR activation could lead to cell dysfunction or apoptosis (Ren et al., 2021). Thus, inhibition of excess ER stress not only reduces oxidative damage but is also a potentially novel therapeutic intervention for cardiac hypertrophy.

Nuclear factor erythroid 2-related factor 2 (NRF2), a transcription factor, reportedly protects against various oxidative stress-induced diseases and has been suggested as a promising therapeutic target for cardiac oxidative damage (Tian et al., 2021a). Increasing evidence has shown that NRF2 activity is limited in a normal cellular microenvironment (Cheng et al., 2021). However, under constant oxidative conditions, NRF2 released in the cytoplasm from inactivated NRF2-Kelch-like ECH associated protein 1 (Keap1) complex promotes the expression of gene subsets correlated with antioxidant responses such as genes encoding heme oxygenase-1 (*HO-1*), oxidoreductase 1 (*NQO1*), and other antioxidant proteins through binding to the antioxidant response elements (ARE) sequences (Nezu and Suzuki, 2021). It has been reported that NRF2 overexpression inhibits left ventricular hypertrophy (LVH) and cardiac remodeling and is a critical factor in maintaining cardiac structural and functional integrity under abnormal stress conditions (Smyrnias et al., 2015). A redox sensor, mTOR, regulates the balance of cellular stress and the SCF/β-TrcP complex composed of the protein Skep1, CUL1, F-box β-TrcP, and the GING finger protein RBX1, which promotes NRF2 proteasomal degradation and regulates intracellular ROS production via mTOR in other stress-induced diseases (Chen et al., 2019). Thus, interventions of NRF2 activity *via* the mTOR pathway may be developed for treating cardiac hypertrophy.

Traditional medicinal herbs have been considered valuable sources for identifying lead compounds and their subsequent refinement into safe and efficacious drugs, such as the antimalarial drug artemisinin (Fu et al., 2021). Particularly, botanical formulations in traditional Chinese medicine (TCM) are usually composed of several types of medicinal compounds or plants, which synergize to achieve holistic therapeutic outcomes (Hu et al., 2019). A novel formulation, Dantonic^®^, containing standardized extracts of *Salvia miltiorrhiza* and *Panax notoginseng* plus borneol, has been approved by the China Food and Drug Administration to treat myocardial and cerebral ischemic injury. The phase III clinical trials of Dantonic^®^ for stable angina pectoris treatment have been completed in the USA (NCT01659580) (Liao et al., 2019a). Tanshinol (DSS, (+) β‐(3,4‐dihydroxy phenyl) lactic acid) is a primary ingredient of an aqueous extract of *S. miltiorrhiza* with pleiotropic properties, which protects the myocardium against ischemia/reperfusion injury (Dai and Zhou, 2015). However, DSS is a hydrophilic molecule with poor solubility in lipidic matrixes, rendering its ability across the cell membrane (Dong et al., 2009). Inspired by the principle of TCM combinatorial formulations, we designed tanshinol borneol ester (DBZ,1,7,7-trimethylbicyclo [2.2.1] heptan-2-yl-3-(3,4-dihydroxy phenyl)-2-hydroxy-propanoate) through chemically combining DSS and borneol (core effective components of Dantonic^®^) (Liao et al., 2019b). Although DBZ reportedly ameliorates lipopolysaccharide (LPS)-induced neuroinflammation and ischemic stroke via Akt/GSK3β/NRF2 pathway in rats (Liao et al., 2020), its functions in cardiac hypertrophy have not yet been studied. Thus, in this project, we investigated the potential effects and mechanisms of DBZ in cardiac hypertrophy caused by chronic pressure overload. Our result suggested that DBZ protected the heart from hypertrophy by inhibiting excess oxidative stress and ER stress.

## Materials and Methods

### Reagents

Pharmacological agents used: angiotensin II (Ang II), chloroquine (CQ), Xanthine oxidase (XO), and rapamycin (Rapa) were purchased from Sigma-Aldrich (St. Louis, MO, United States). Rhodamine phalloidin was acquired from Cytoskeleton (Denver, CO, United States). Mito-TEMPO and 4-PBA were obtained from Sigma-Aldrich (Sigma, St. Louis, MO, United States). Antibodies against LC3A/B, ATG5, p62, mTOR, p-mTOR, ATG5, ATG7, Beclin 1, PERK, XBP1, CHOP, β-actin, and GAPDH were purchased from Cell Signaling Technology (Boston, MA, United States). GRP78, GSK3β, p-GSK3β (Ser9), β-TrcP, and NRF2 were purchased from Abcam (Cambridge, MA, United States). NOX2, NOX4, and HO-1 were provided by Proteintech (Wuhan, China).

### Transverse-Aortic Constriction-Induced Left Ventricular Hypertrophy Model and Tanshinol Borneol Ester Treatment

The animals were subjected to left lateral thoracotomy and TAC as previously described (Han et al., 2021). Briefly, rats were anesthetized with 0.5%–2% isoflurane in 100% oxygen to keep the oxygen saturation and blood pH within normal ranges. All experimental protocols were approved by the Animal Research Center of Zheng Zhou University and conformed to the Guide for the Care and Use of Laboratory Animals published by the US National Institutes of Health (NIH Publication No. 8523). Male Sprague-Dawley rats (150–180 g), obtained from Beijing Weitong Lihua Experimental Animal Technology Co., Ltd., were housed in individual cages under a 12:12 h light/dark cycle at room temperature, fed with a standard laboratory rodent chow, and provided water *ad libitum*. The hearts were exposed at the third intercostal space and isolated the transverse aorta; a 4/0 silk suture was drawn under the transverse aorta with a 22 G needle and tied firmly to the aorta. Then, the needle was removed within 5 s. The sham group was operated in the same way but without constriction. Doppler echocardiography was performed to further confirm the efficacy of the TAC procedure 6 h post-surgery. The sham and TAC groups were randomized into vehicle and DBZ-treated subgroups, respectively, as follows: sham + vehicle (*n* = 10); sham + DBZ (*n* = 10); TAC + vehicle (*n* = 10); TAC + DBZ (*n* = 10). DBZ (dissolved using 0.2% (w/v) poloxamer 188 solution (BASF, Ludwigshafen, Germany)) was administered at the dose of 20 mg/kg/day by intraperitoneal injections for 8 weeks according to previous studies (Liao et al., 2019b; Liao et al., 2020). Two rats died of aortic arch rupture on days 28 and 36 after the surgery in the TAC group, whereas DBZ did not increase the mortality of rats in the DBZ group. All animals were euthanized as required, and the heart tissues were preserved for further experiments.

### Echocardiography

Echocardiography was performed at 8 weeks post-operation using the Vevo 2100 system (VisualSonics, Toronto, Canada). Rats (*n* = 10) were anesthetized in the supine position on a platform with body temperature at about 37°C. Parasternal long-axis pictures of the left ventricle (LV) were evaluated at the papillary muscle level using a 10 MHz probe. Two-dimensional (2D) guided M-mode echocardiography was recorded for more than five cardiac cycles to measure parameters such as intraventricular septal thickness diastole (IVSd), LV internal dimensions systole (LVIDs), LV posterior wall dimensions diastole (LVPWd), and LV ejection fraction (EF), LV fractional shortening (FS).

### Histopathological Staining

Hearts were collected 8 weeks post-operation, immediately fixed with 4% paraformaldehyde, and cut into 5-μm thick slices. The cross-section of the LV was evaluated using hematoxylin and eosin (HE) staining. Fibrotic areas of the myocardium were evaluated using Masson’s trichrome staining. Autophagy was determined using p62 immunofluorescence. The photomicrographs were digitalized and analyzed using ImageJ (NIH, Bethesda, United States) and Image-pro Plus version 6.0 (Media Cybernetics, Silver Spring, MD, United States).

### Neonatal Rat Cardiomyocytes Isolation and Culture

NRCMs were isolated as described previously (Wang et al., 2020). Briefly, hearts were cut into small pieces less than 1 mm^3^ in size. Excess blood was washed using phosphate-buffered saline (PBS), following which the heart pieces were incubated with 0.08% trypsin and collagenase for 10 min at 37°C for enough cycles. The digested cell/tissue mixture was filtered and centrifuged, then resuspended in Dulbecco’s modified Eagle’s medium (DMEM, Biological Industries, Kibbutz Beit Haemek, Israel) supplemented with 10% fetal bovine serum and 100 units/ml penicillin and streptomycin. Non-adherent cardiomyocytes were collected after 2 h and cultured in a medium containing 0.1 mM bromodeoxyuridine. Rapamycin (0.5 mM) was added and incubated for 2 h, followed by treated with DBZ (10 μM) and angiotensin II (20 μM) for 24 h.

### Transmission Electron Microscopy

Heart tissues were cut into 1 mm^3^ cubes and fixed overnight using cold 2.5% glutaraldehyde in PBS. Then post-fixed in 1% OsO_4_ (in water) and embedded in epoxy resin. The ultrathin sections were stained with uranium and lead salts and analyzed using a transmission electron microscope (JEM-1200EX, Japan).

### Immunofluorescence Staining

NRCMs (1 × 10^5^ cells/cm^2^) were seeded on coverslips and incubated with Ang II (20 μM, 24 h). Then, fixed with 4% paraformaldehyde for 10 min after washed, and permeabilized with 0.1% Triton X-100. Next, the slips were incubated in blocking solution for 1 h and then with primary antibody overnight at 4°C. Finally, cells were incubated with secondary antibody conjugated with Alexa Fluor^®^ 488 or 594. A confocal microscope (LSM 800; Carl Zeiss Inc, Oberkochen, Germany) was used to capture images.

### Determination of Oxidative Stress

Rats were sacrificed, the hearts were washed with cold PBS, embedded in the OCT compound, and frozen. Then the tissues were cut into 5 μm and thawed. The diluted DHE staining solution (Sigma, United States) was used to incubate different sections for 30 min without light. Then, cold PBS was used to rinse the sections twice.

Intracellular ROS levels were detected using a commercial kit (Beyotime, Shanghai, China). Briefly, the NRCMs were seeded in 96-well plates, incubated with DCFH-DA (10 µM, a fluorescent ROS probe) at 37°C for 30 min, and washed three times. The fluorescence intensities at 488 and 525 nm were detected using a luminometer (Synergy HT, BioTek, United States).

### Reverse Transcription-Quantitative PCR

The mRNA levels of the embryonic genes, *Nppa* and myosin heavy chain (*Myh7*), were determined using RT-qPCR. Briefly, RNAs were isolated using TRIzol reagent (Thermo Fisher, Waltham, MA, United States). RT-qPCR was performed using cDNA reverse transcribed using the PrimeScriptRT detection kit (Takara Biotechnology, Tokyo, Japan) and detected using the ABI Prism 7500 sequence detection system (Thermo Fisher, Waltham, MA, United States). The amplification conditions and cycles were performed according to the manufacturer’s instructions. The threshold cycle (Ct) was defined within the exponential phase. The relative gene expression among the treatments was calculated using the following equation: relative gene expression = 2^−(ΔCt sample−ΔCt control)^.

### Western Blotting

Myocardial extracts were lysed using SDS lysis buffer (1% SDS, 50 mM Tris-HCl) and examined using Western blotting. In total, 50 μg myocardial extracts were separated via SDS-polyacrylamide gel electrophoresis, transferred, and blocked. Then membranes were incubated with each primary antibody, washed thrice with TBST, and incubated for another 2 h at 37°C with secondary antibodies (1:10,000), followed by washing thrice with TBST, 10 min each time. Protein bands were visualized using an ECL Western blotting detection system (KeyGen).

### Statistical Analysis

Analyses were performed using the *SPSS* 14.0 (SPSS Inc.). All data were expressed as mean ± standard deviation (SD). Comparisons between two groups were assessed using a Student’s t-test. One-way analysis of variance (ANOVA) was performed to compare multiple groups at a time point. *p* < .05 was considered statistically significant.

## Results

### Tanshinol Borneol Ester Improved Cardiac Function and Attenuated Cardiac Remodeling in Rats With Transverse-Aortic Constriction

**FIGURE 1:**
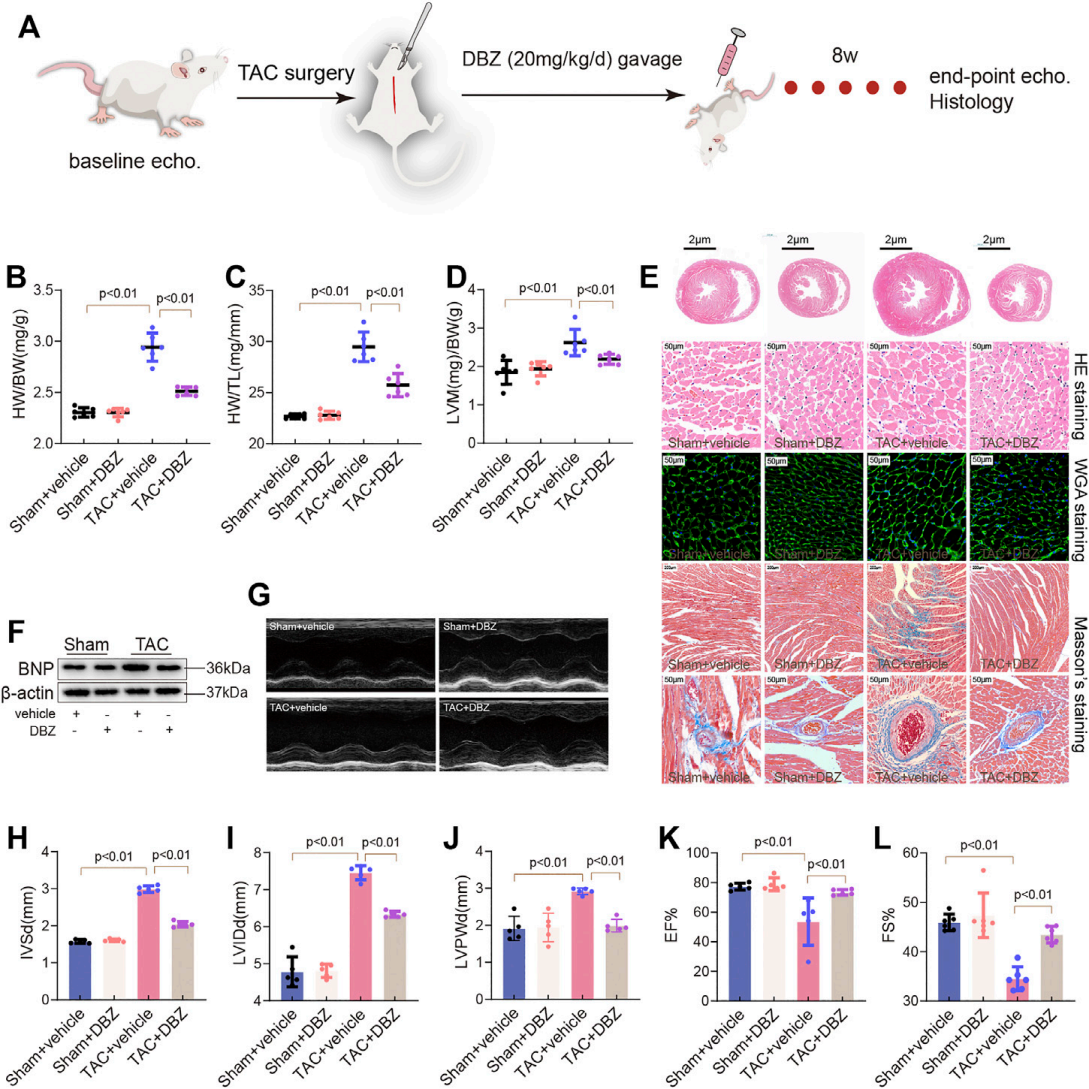
DBZ improves cardiac function and attenuates cardiac remodeling in TAC-induced cardiac hypertrophy. **(A)** Flowchart of study protocol. **(B–D)** The ratios of heart weight/body weight, heart weight/tibia length, and left ventricular mass/body weight of rats (*n* = 10) in the 8th week. **(E)** Representative transverse sections of H&E staining (first row; scale bar = 2 μm), Macroscopic view of H&E staining (second row; scale bar = 50 μm), WGA staining (third row; scale bar = 50 μm) and Masson’s staining (fourth and fifth row; scale bar = 200 and 50 μm) from heart transverse sections. *n* = 4–6. **(F)** Representative images of Western blotting assay of BNP. **(G–L)** Representative M-mode images of the four groups and quantification of interventricular septum diastolic dimension (IVSd), LV internal dimension diastole (LVIDd), and LV posterior wall thickness diastole (LVPWd), ejection fraction (EF) and fractional shortening (FS). *n* = 6. Results are expressed as means ± SD. Statistical analyses were performed by one-way ANOVA followed by Bonferroni’s *post-hoc* test.

To understand the beneficial effects of DBZ in cardiac hypertrophy, we first analyzed whether DBZ could attenuate the hypertrophic response induced by long-term pressure overload. Rats were subjected to TAC surgery, and 20 mg/kg DBZ was administered by intraperitoneal injection once a day for 8 weeks, according to previous studies (Liao et al., 2019b; Liao et al., 2020; Figure 1A). Rats without TAC surgery did not show any alterations in cardiac structure or function with or without DBZ treatment. TAC surgery resulted in higher ventricular wall transformation and inferior cardiac function than sham surgery. However, rats treated with DBZ showed lesser indications of cardiac remodeling than untreated rats, as shown by a smaller increase in heart weight (HW)/body weight (BW) ratio, heart weight (HW)/tibia length (TL) ratio, and left ventricular mass (LVM)/BW (Figures 1B–D). Consistently, histological detection using HE and wheat germ agglutinin (WGA) staining showed that DBZ markedly ameliorated cardiomyocyte hypertrophy induced by TAC (Figure 1E; Supplementary Figure S1A). Furthermore, the increase in *Nppa* and *Myh7* expression (associated with cardiac hypertrophy) after TAC was significantly attenuated in DBZ-treated rats (Supplementary Figures S1B,C). In addition, the results of Western blotting also indicated that DBZ inhibited the increase in brain natriuretic peptide levels (Figure 1F; Supplementary Figure S1D).

Compared to that observed in the sham group, echocardiography showed an increase in LVM, IVSd, LVID, and LVPWd in the TAC group. In contrast, the DBZ treatment group showed lesser remodeling of the ventricular wall (Figures 1G–J; Supplementary Figure S1E). In addition, DBZ elevated EF% and FS%, indicating that DBZ treatment resulted in better cardiac output (Figures 1K–L).

The maladaptive response of cardiomyocytes leading to cardiac remodeling manifests as an increase in apoptosis and cardiac fibrosis (Wang et al., 2020). After 8 weeks, TAC induced significant fibrosis in myocardial tissue or around the perivascular space; however, surprisingly, excessive fibrosis was not observed in the DBZ treatment group (Figure 1E; Supplementary Figure S1F). Furthermore, DBZ treatment also inhibited the expression of collagen-related genes (procollagen I and procollagen III) (Supplementary Figures S1G,H). These observations indicated that DBZ improves cardiac function and reduces chamber enlargement, which may delay the progression of cardiac hypertrophy to heart failure.

### Tanshinol Borneol Ester Inhibited Ang II-Induced Cardiomyocyte Hypertrophy

**FIGURE 2:**
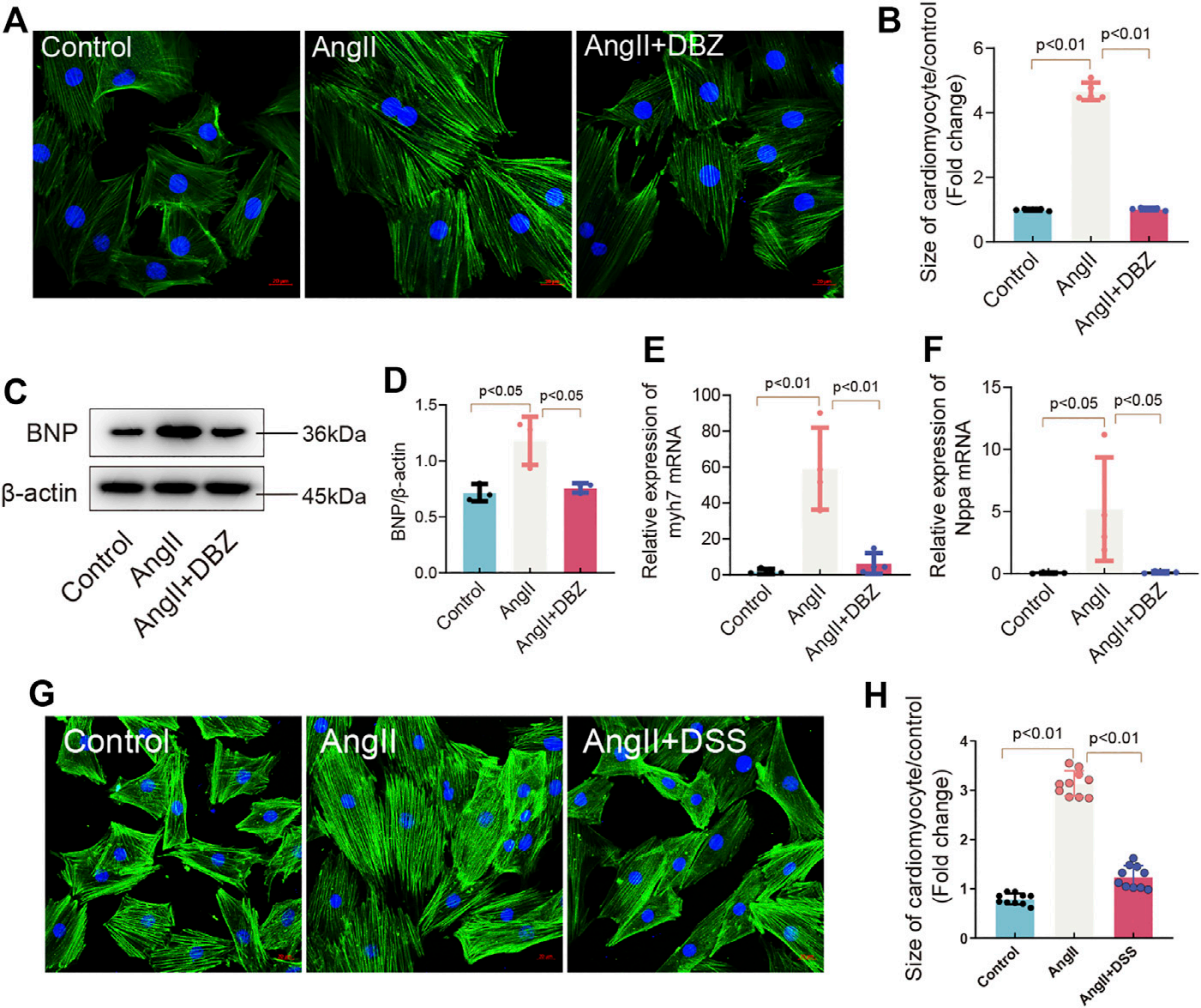
DBZ inhibited cardiomyocyte hypertrophy. **(A,B)** Rhodamine phalloidin staining of NRCMs and quantification of the size of cardiomyocytes. Scale bars represent 20 μm. *n* = 10. **(C,D)** Representative Western blotting assay and quantification of BNP expression. *n* = 3. **(E,F)** Real-time PCR quantification of relative mRNA expression level of myh7 and Nppa. *n* = 4. **(G,H)** Rhodamine phalloidin staining of NRCMs and quantification of the size of cardiomyocytes. Scale bars represent 20 μm. *n* = 10. Results are expressed as means ± SD. Statistical analyses were performed by one-way ANOVA followed by Bonferroni’s *post-hoc* test.

To determine whether DBZ can protect cardiomyocytes against stress-induced hypertrophy, the primary cultured NRCMs were extracted and cultured in a medium supplemented with Ang II for 24 h. Concentrations of DBZ (10 μM) that were shown to be safe were used (Supplementary Figure S2A) (Liao et al., 2019b; Liao et al., 2020). Cell size was measured using fluorescein isothiocyanate-phalloidin staining. The results showed that DBZ attenuated the increase in cardiomyocyte size induced by Ang II after 24 h of incubation (Figures 2A,B) and suppressed BNP expression, which was elevated after Ang II exposure (Figures 2C,D). In addition, DBZ markedly attenuated the increase in Ang II-induced transcription of *Nppa* and *Myh7*, markers of myocardial hypertrophy (Figures 2E,F). Danshensu is identified as the major end metabolic product of DBZ, so we detected whether DSS exerts the same effects in cardiac hypertrophy as DBZ. The data showed that DSS also decreased the cell size induced by Ang II, further demonstrating the anti-hypertrophy function of DBZ is DSS-dependent (Figures 2G,H). These results indicated that DBZ could inhibit Ang II-induced NRCMs hypertrophy.

### Tanshinol Borneol Ester Attenuated the Oxidative Stress Induced by Transverse-Aortic Constriction and Angiotensin II

**FIGURE 3:**
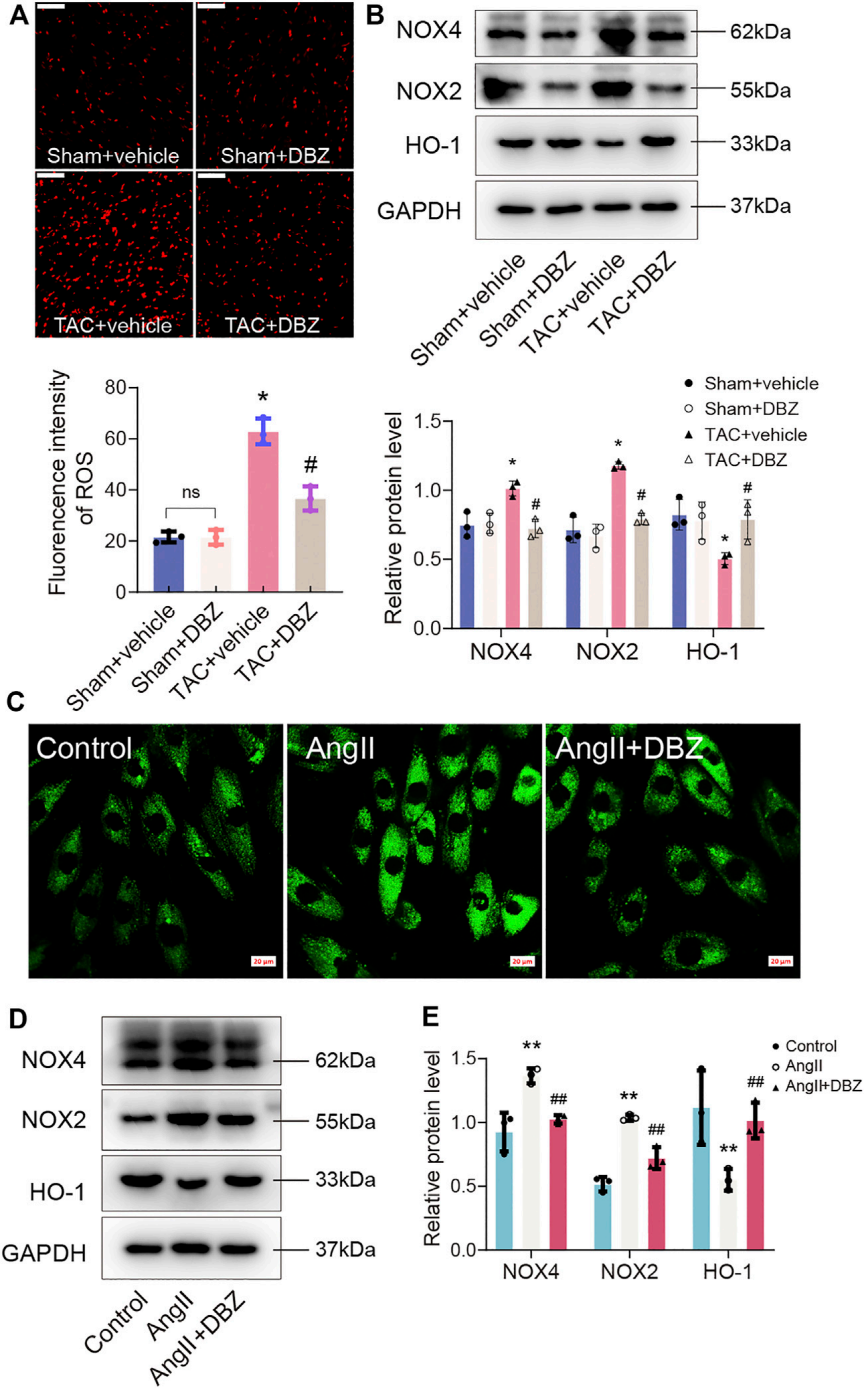
DBZ attenuated the oxidative stress. **(A)** Representative images and quantification of DHE staining in transverse sections and quantification. *n* = 3. **(B)** Protein levels of NOX4, NOX2 and HO-1 were determined by Western blotting assay and quantified by densitometry. *n* = 3. **(C)** The ROS generation measured using DCFH-DA. *n* = 3. Scale bar = 20 μm. **(D,E)** Representative Western blotting assay and quantification of NOX4, NOX2 and HO-1 expression. *n* = 3. *p < .05 compared with the sham group, ^#^ *p* < .05 compared with the TAC group, **p < .05 compared with the control group, ^##^ *p* < .05 compared with the Ang II group. Results are expressed as means ± SD. Statistical analyses were performed by one-way ANOVA followed by Bonferroni’s *post-hoc* test.

Oxidative stress contributes to the development of cardiac hypertrophy. Moreover, DBZ reportedly exhibits antioxidant activity (Liao et al., 2020). Hence, we evaluated the antioxidative effects of DBZ in rats and NRCMs. First, we assessed ROS production in the myocardium using DHE staining. As expected, our results showed that the myocardial ROS level was higher in the group with TAC surgery than in the sham group. However, DBZ restricted ROS generation after TAC surgery (Figure 3A). Western blotting showed that NOX2 and NOX4 expression (mediating the generation of ROS) was significantly reduced, whereas that of HO-1 increased after DBZ treatment (Figure 3B). Quantitative analysis also showed enhancement in myocardial superoxide dismutase-1 (SOD-1) and glutathione peroxidase (GPx) level, whereas the malondialdehyde (MDA) level was reduced in the DBZ-TAC group (Supplementary Figures S2B–D).

Similar results were obtained in cardiomyocytes induced by Ang II. We evaluated the ROS production inside the cell and mitochondria using DCFH-DA and MitoSox fluorescence. The data showed that DBZ inhibited total ROS production and mitochondrial ROS generation (Figure 3C; Supplementary Figures S2E,F). In addition, NOX2 and NOX4 were downregulated, whereas HO-1 was upregulated, similar to that observed *in vivo* (Figures 3D,E). Taken together, all the data mentioned above suggest that DBZ reduces ROS accumulation by inhibition of ROS generation through NOX upregulation and scavenging ROS through the upregulation of mitochondrial antioxidant enzymes.

### Tanshinol Borneol Ester Attenuated Endoplasmic Reticulum Stress Induced by Transverse-Aortic Constriction and Angiotensin II

**FIGURE 4:**
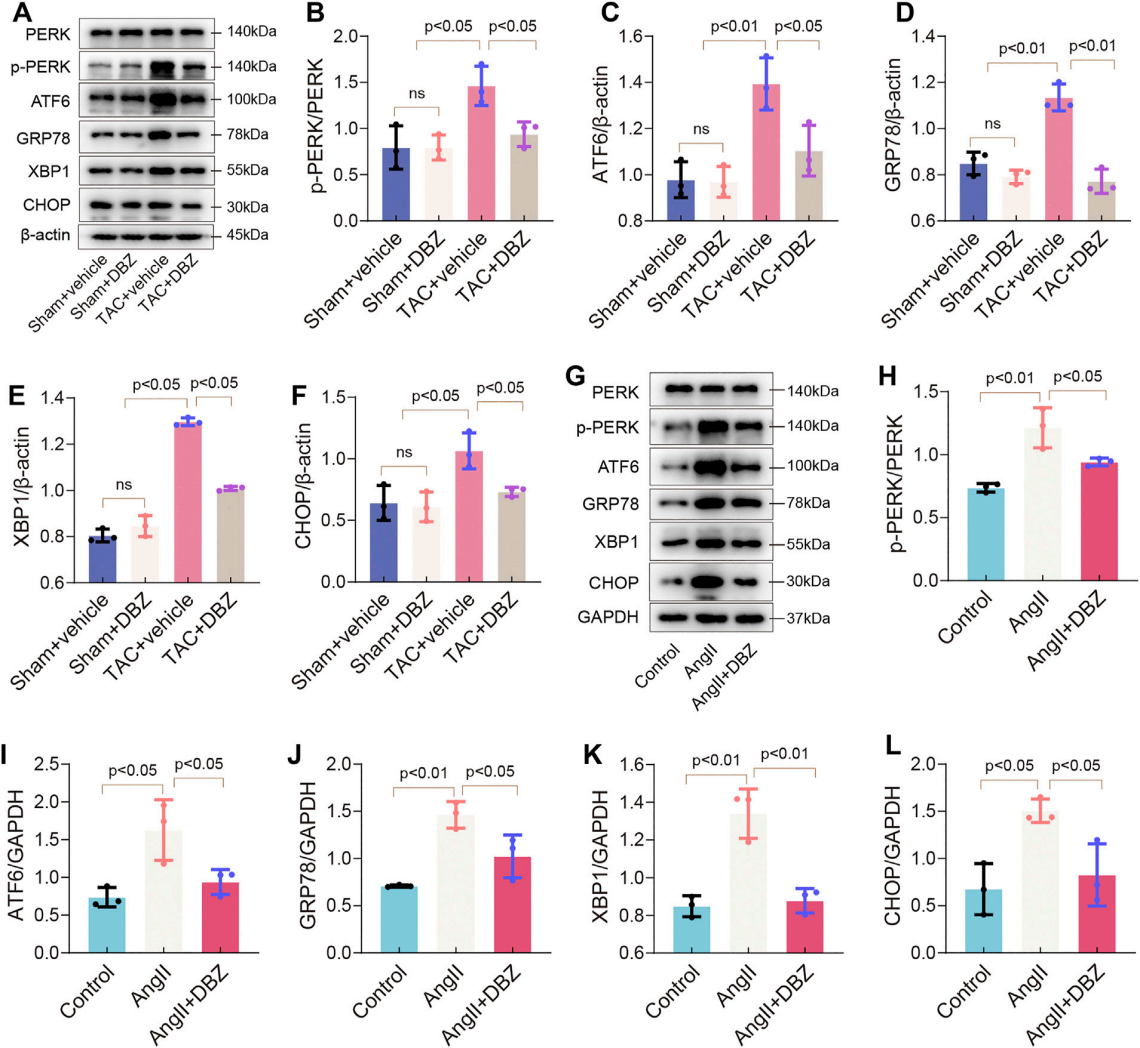
DBZ attenuated ER stress. **(A–F)** Protein levels of PERK, p-PERK, GRP78, ATF6, CHOP, XBP1 were determined by Western blotting assay and quantified by densitometry. *n* = 3. **(G–L)** Protein levels of PERK, p-PERK, GRP78, ATF6, CHOP, XBP1 were determined by Western blotting assay and quantified by densitometry. *n* = 3. Results are expressed as means ± SD. Statistical analyses were performed by one-way ANOVA followed by Bonferroni’s *post-hoc* test.

ER and oxidative stress crosstalk and are known to cause cardiomyocyte dysfunction (Ceylan-Isik et al., 2013). Hence, we assessed the effects of DBZ on ER stress. As shown in Figures 4A–F, the expression of TAC-induced ER stress markers including p-PERK, GRP78, ATF6, XBP1, and CHOP were attenuated after DBZ treatment; DBZ also considerably inhibited ER stress in the presence of Ang II (Figures 4G–L). These results suggested that DBZ not only inhibited ROS production but also reduced intracellular ER stress.

### Tanshinol Borneol Ester Inhibited Stress-Induced Autophagy

**FIGURE 5:**
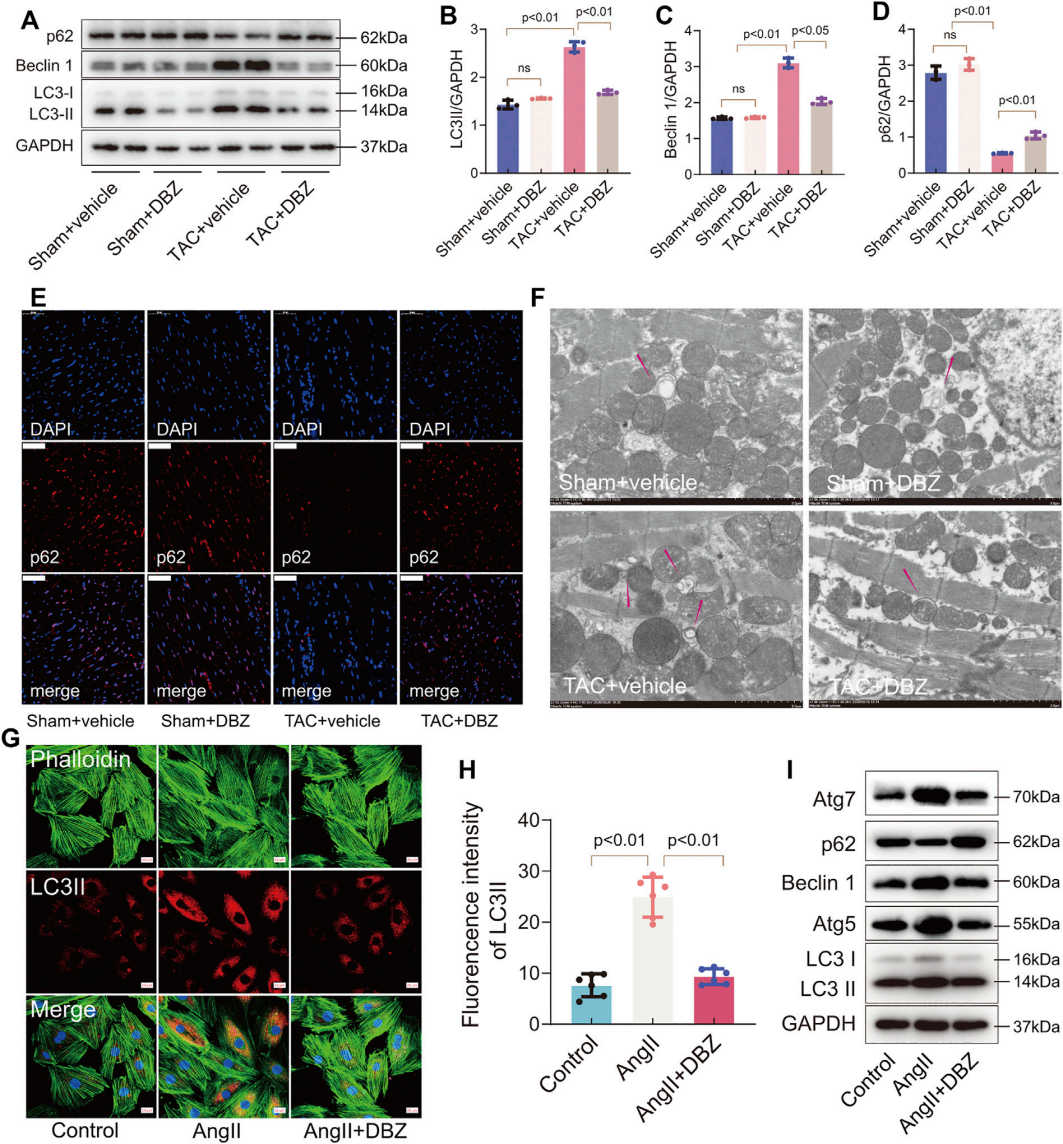
DBZ inhibited stress-induced autophagy. **(A–D)** Representative Western blotting assay and quantification of LC3, p62, and Beclin1 expression. *n* = 3. **(E)** Immunofluorescence staining of p62 in cardiac tissues. Scale bars = 50 μm. *n* = 3. **(F)** Representative electron micrographs of autophagic vacuoles in cardiomyocytes of rats with various treatments. Scale bars = 2 μm. *n* = 3. **(G,H)** Immunofluorescence staining and quantification of LC3-II in NRCMs. *n* = 5. Scale bars = 20 μm. **(I)** Representative Western blotting assay of Atg7, p62, Beclin1, Atg5 and LC3 expression. Results are expressed as means ± SD. Statistical analyses were performed by one-way ANOVA followed by Bonferroni’s *post-hoc* test.

Autophagy is an evolutionarily conserved process that degrades dysfunctional cellular components and plays important roles in cardiac hypertrophy and heart failure (Ljubojević-Holzer et al., 2021). The crosstalk between autophagy, ER stress, and oxidative stress is involved in the progression of cardiac hypertrophy (Pires Da Silva et al., 2020). Therefore, we used LC3, Beclin 1, ATG7, ATG5, and p62 to monitor autophagy by Western blotting and immunofluorescence. The results showed that 8 weeks of TAC enhanced LC3-II and Beclin 1 expression but inhibited p62 expression, indicating that autophagy was activated under maladaptive stress. However, DBZ inhibited the elevation of autophagy in hypertrophic hearts (Figures 5A–D). Similar results were also obtained using p62 immunofluorescence and TEM in the rat model of hypertrophy (Figures 5E,F; Supplementary Figure S3A).

Similarly, immunofluorescence staining revealed that DBZ inhibited LC3-II expression in NRCMs (Figures 5G,H). The levels of LC3, Beclin 1, ATG7, ATG5, and p62 related to autophagy detected by Western blotting suggested that DBZ inhibited autophagy (Figure 5I; Supplementary Figures S3B–F). Although autophagy is usually regarded as a double‐edged sword in cardiac hypertrophy, the levels of the participating proteins in heart tissue may determine the outcome. Here, we observed that DBZ treatment might ameliorate cardiac hypertrophy by inhibiting excessive autophagy.

### Tanshinol Borneol Ester Ameliorated Myocardial Apoptosis in Rats and Cells

**FIGURE 6:**
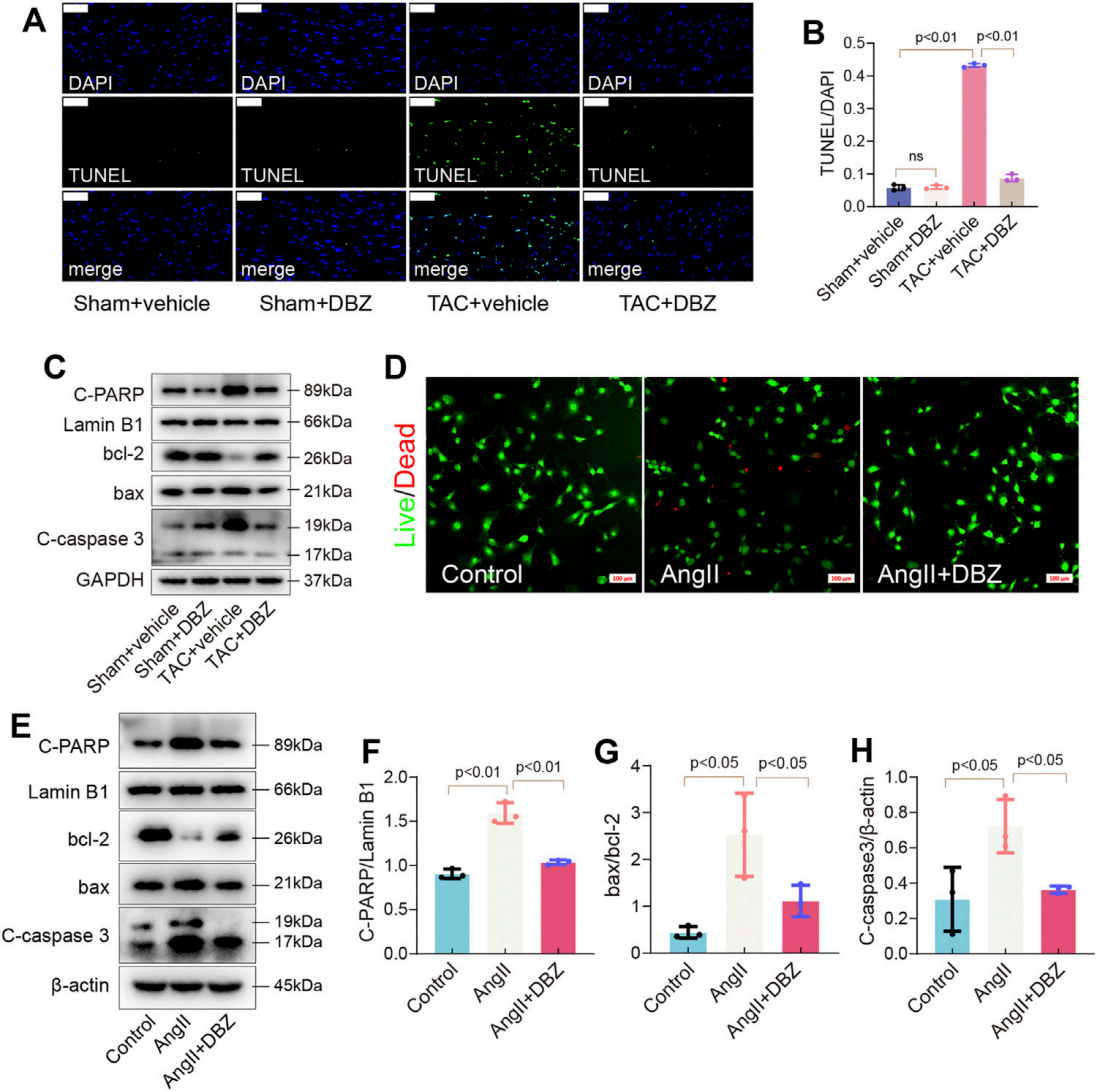
DBZ ameliorated apoptosis of cardiomyocytes. **(A,B)** The apoptosis level detected by TUNEL staining and quantification. Scale bars = 50 μm. *n* = 3. **(C)** Representative Western blotting assay of C-PARP, bcl-2, bax and C-caspase3 expression. **(D)** Representative *Live/Dead* staining images. Scale bars = 100 μm. **(E–H)** Representative Western blotting assay and quantification of C-PARP, bcl-2, bax and C-caspase3. *n* = 3. Results are expressed as means ± SD. Statistical analyses were performed by one-way ANOVA followed by Bonferroni’s *post-hoc* test.

Imbalance in oxidative stress, ER stress, or both can lead to apoptosis. Here, pressure overload due to TAC for 8 weeks significantly increased the number of TUNEL-positive cardiomyocytes in sections of the vehicle-TAC group, while DBZ reversed these changes (Figures 6A,B). Furthermore, TAC triggered the expression of apoptosis-related proteins, including Bax, Cleaved-caspase 3, and Cleaved-poly (ADP-ribose) polymerase (PARP), and inhibited BCL-2 expression, which was reversed by DBZ treatment (Figure 6C; Supplementary Figures S4A–C).

In addition, detecting apoptosis in NRCMs using dead/live staining revealed that DBZ reduced the number of apoptotic cells induced by Ang II (Figure 6D; Supplementary Figure S4D). The expression of apoptosis-related proteins was similar to that observed in rats (Figures 6E–H).

### Intracellular Reactive Oxygen Species Contributed to Endoplasmic Reticulum Stress, Autophagy, and Apoptosis

**FIGURE 7:**
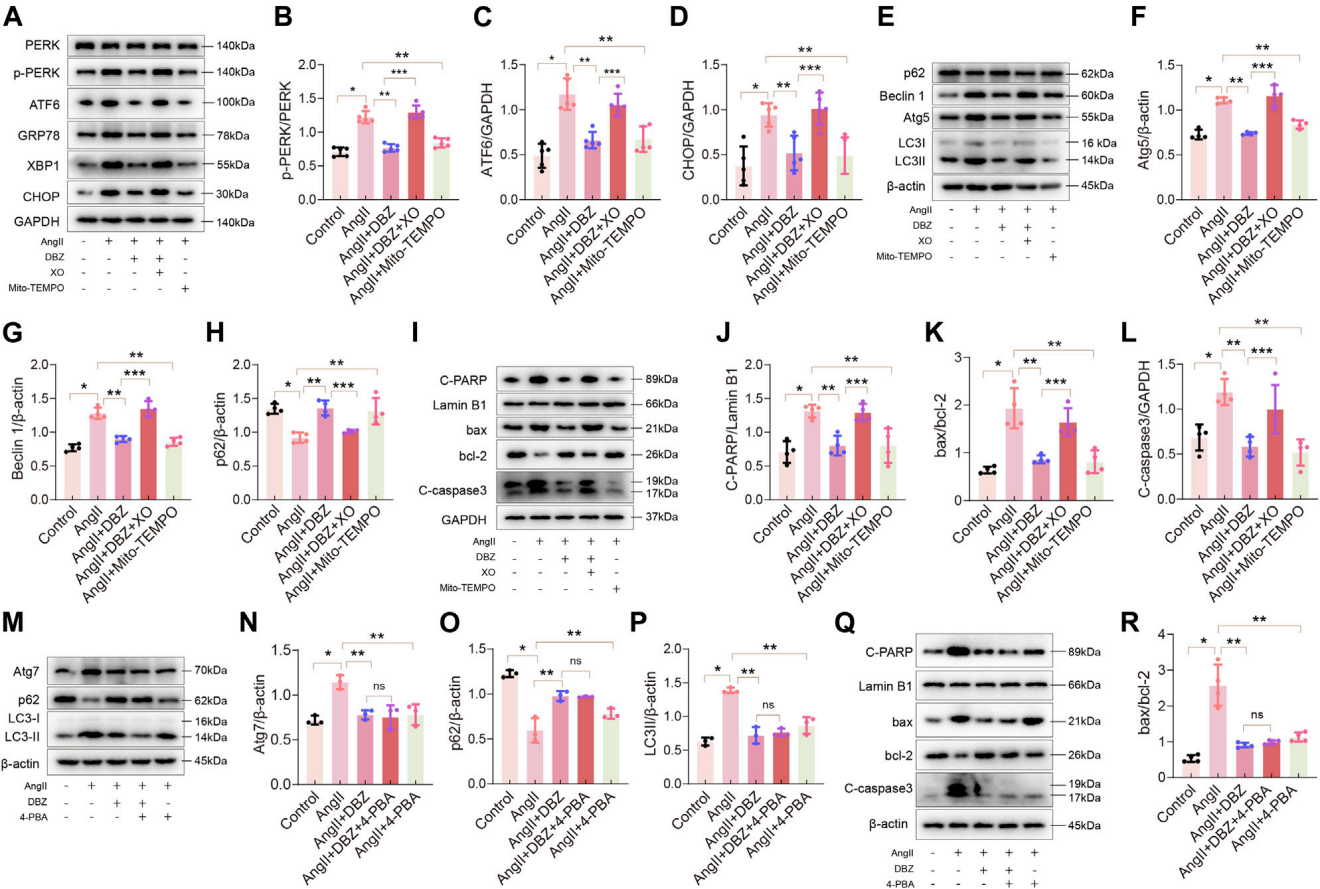
Oxidative stress contributed to ER stress, autophagy and apoptosis. **(A–D)** Representative Western blotting assay and quantification of PERK, p-PERK, ATF6, CHOP, GRP78 and XBP1 expression. *n* = 4–5. **(E–H)** Representative Western blotting assay and quantification of Atg5, Beclin1, p62 and LC3 expression. *n* = 4. **(I–L)** Representative Western blotting assay and quantification of C-PARP, bax, bcl-2 and C-caspase3 expression. *n* = 4. **(M–P)** Representative Western blotting assay and quantification of Atg7, p62 and LC3 expression. *n* = 3. **(Q–R)** Representative Western blotting assay and quantification of C-PARP, bax, bcl-2 and C-caspase3 expression. *n* = 4. *p < .05 compared with the Control group, **p < .05 compared with the Ang II group, ***p < .05 compared with the Ang II + DBZ group. Results are expressed as means ± SD. Statistical analyses were performed by one-way ANOVA followed by Bonferroni’s *post-hoc* test.

To further confirm whether stress-induced ROS accumulation was responsible for ER stress and activation of autophagy in cardiac hypertrophy, we used the antioxidant, Mito-TEMOP (10 µM for 24 h), and the oxidation inducer, XO (50 mU for 24 h), to culture NRCMs, followed by detection of GRP78 and other ER stress signal markers using Western blotting. Mito-TEMPO obliterated Ang II-induced increase in the expression of p-PERK, GRP78, ATF6, XBP1, and CHOP (Figures 7A–D; Supplementary Figures S5A,B). Consistently, Mito-TEMPO also inhibited autophagy and protected against apoptosis, as shown by the inhibition of the expression of LC3-II, ATG5, Beclin 1, C-PARP, Bax, and C-caspase3, as well as the increase in p62 and BCL-2 activity (Figures 7E–L; Supplementary Figures S5C). Expectedly, XO reversed the effects of DBZ in ER stress, autophagy, and apoptosis. Next, we used an ER stress inhibitor, 4-phenylbutyrate (4-PBA), to detect whether inhibition of ER stress prevented Ang II-induced intracellular autophagy and cardiomyocyte death. Results showed that 4-PBA abolished Ang II-elicited autophagy and apoptosis similar to DBZ, although the co-application of DBZ and 4-PBA did not further reduce the level of autophagy or apoptosis; nonetheless, we believe that ER stress considerably contributes to autophagy and apoptosis (Figures 7M–R; Supplementary Figures S5D,E). Furthermore, to specifically understand the role of autophagy in cardiac hypertrophy, we exposed cells to rapamycin (Rapa) or chloroquine (CQ), which increased or inhibited autophagy. Results showed that Rapa abolished the DBZ-mediated inhibition of autophagy but did not affect its antioxidative and antiapoptotic properties (Supplementary Figures S5F–P). Interestingly, autophagy inhibition did not protect against cardiac hypertrophy. These results indicated that autophagy is not the main mechanism via which DBZ prevents cardiac hypertrophy, and DBZ improved cardiac function primarily by reducing the damage of oxidative stress.

### Nuclear Factor Erythroid 2-Related Factor 2 Activation was Critical for Antioxidation and for Inhibiting Endoplasmic Reticulum Stress of Tanshinol Borneol Ester

**FIGURE 8:**
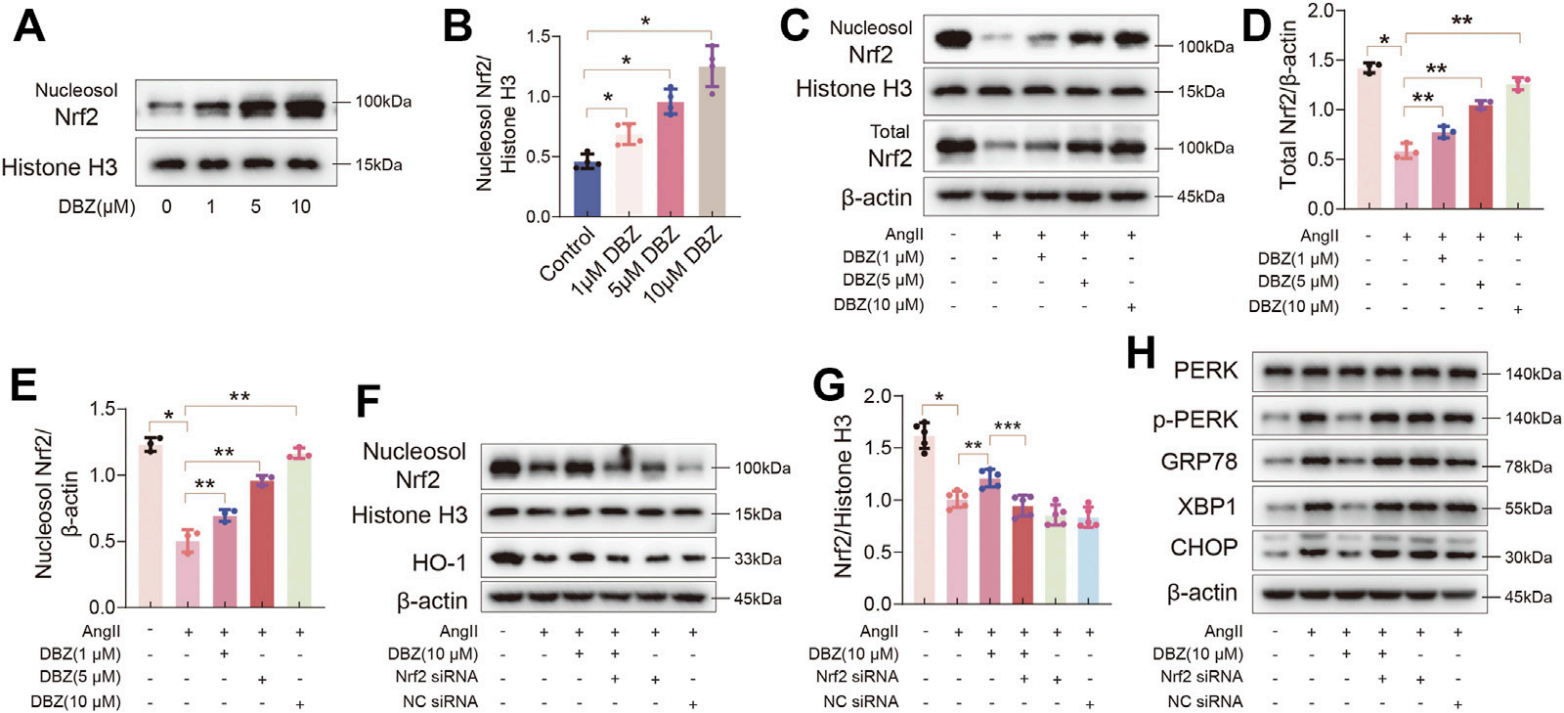
Nrf2 activation was critical for the antioxidant and inhibiting ER stress of DBZ. **(A,B)** Representative Western blotting assay and quantification of nucleosol Nrf2 expression with DBZ treatment. *n* = 4. **(C–E)** Representative Western blotting assay and quantification of nucleosol and total Nrf2 expression. *n* = 3. **(F,G)** Cells transfected with Nrf2 siRNA or NC siRNA, protein levels of nucleosol Nrf2 were determined by Western blotting assay and quantified by densitometry. *n* = 5. **(H)** Representative Western blotting assay of PERK, p-PERK, GRP78, XBP1 and CHOP expression. *p < .05 compared with the Control group, **p < .05 compared with the Ang II group, ***p < .05 compared with the Ang II + DBZ group. Results are expressed as means ± SD. Statistical analyses were performed by one-way ANOVA followed by Bonferroni’s *post-hoc* test.

NRF2 is a master regulator of the expression of antioxidant enzymes, such as HO-1. Thus, we detected whether DBZ could stabilize and activate NRF2 in NRCMs. Western blotting results showed that DBZ enhanced the NRF2 nuclear accumulation in a concentration-dependent manner (Figures 8A,B). Furthermore, DBZ promoted the DNA-binding activity of NRF2 in NRCMs (Supplementary Figure S6A). Interestingly, we found that DBZ not only promoted the nuclear accumulation of NRF2 in Ang II-damaged cells but also increased the total protein level of NRF2 (Figures 8C–E).

Next, we knocked down *NRF2* using siRNA to verify whether the antioxidant capacity of DBZ was mediated by increasing NRF2 nuclear accumulation. As shown in Figures 8F–H and Supplementary Figure S6B, Western blot analysis showed that DBZ-mediated increase in the expression of NRF2 and HO-1 in Ang II-treated cells was considerably lowered by NRF2 siRNA transfection. Furthermore, the levels of ER stress markers were elevated when *NRF2* was knocked down (Supplementary Figures S6C–F). The empty plasmid control (NC) did not significantly affect the levels of oxidative stress and ER stress. These results suggested that Nrf2, especially its accumulation in the nucleus, was important for preventing cardiac hypertrophy of DBZ.

### Tanshinol Borneol Ester Inhibited the Degradation of Nuclear Factor Erythroid 2-Related Factor 2 Through the mTOR/β-TrcP Pathway

**FIGURE 9:**
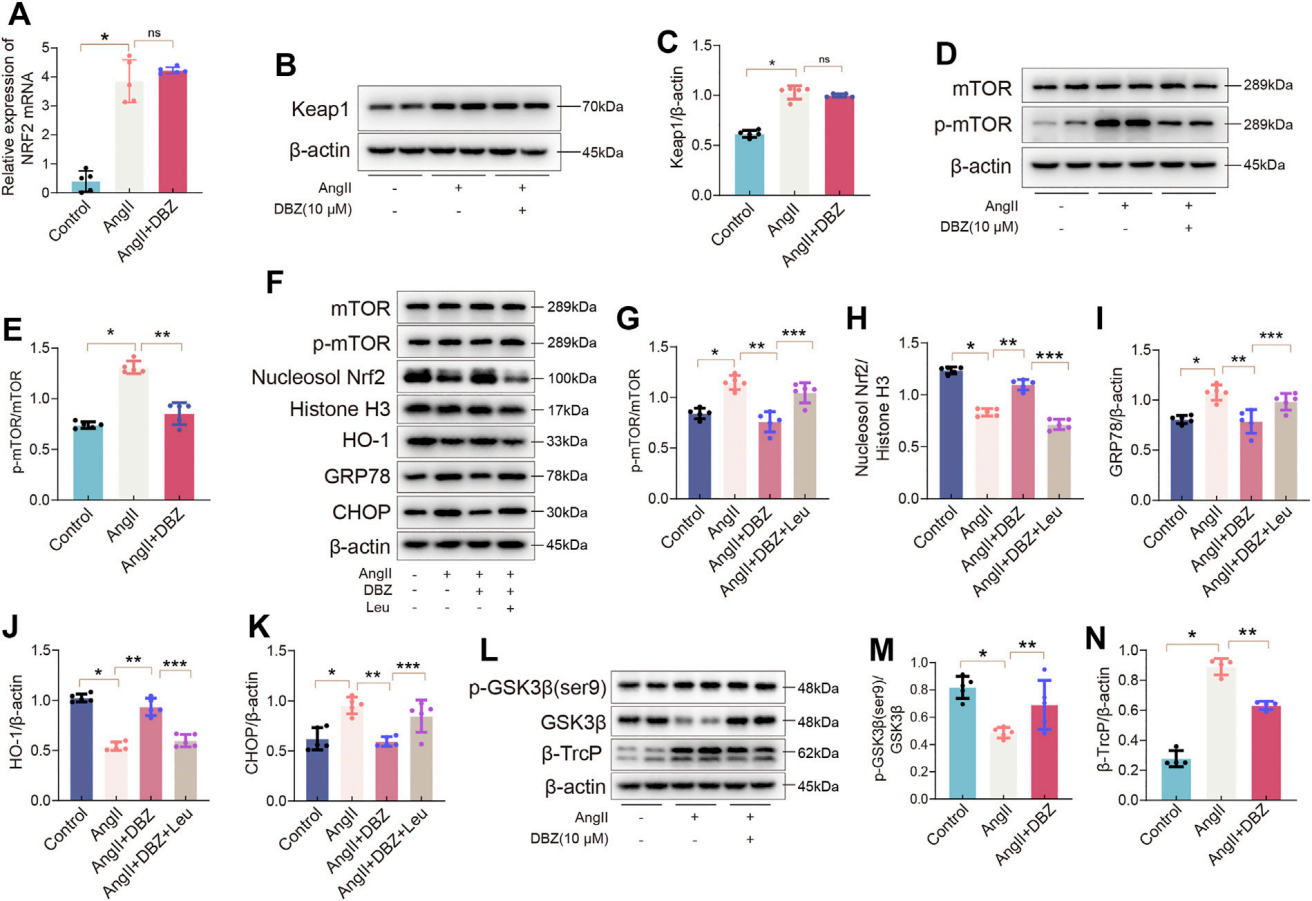
DBZ inhibited the degradation of Nrf2 via the mTOR/β-TrcP pathway. **(A)** Real-time PCR quantification of relative mRNA expression level of Nrf2. *n* = 5. **(B,C)** Representative Western blotting assay and quantification of Keap1. *n* = 5. **(D,E)** Representative Western blotting assay and quantification of p-mTOR and mTOR. *n* = 5. **(F–K)** Representative Western blotting assay and quantification of mTOR, p-mTOR, nucleosol Nrf2, Histone H3, HO-1, GRP78, and CHOP expression. *n* = 5. **(L–N)** Representative Western blotting assay and quantification of p-GSK3β(ser9), GSK3β, and β-TrcP expression. *n* = 4. *p < .05 compared with the Control group, **p < .05 compared with the Ang II group, ***p < .05 compared with the Ang II + DBZ group. Results are expressed as means ± SD. Statistical analyses were performed by one-way ANOVA followed by Bonferroni’s *post-hoc* test.

The mechanism via which DBZ regulated NRF2 levels was investigated. We used RT-PCR to determine whether the increase in NRF2 level was transcriptionally regulated. As shown in Figure 9A, the relative expression of *NRF2* mRNA was not altered with DBZ treatment, indicating that the increased content of NRF2 protein was not due to an increase in transcription. This outcome described above indicated that the DBZ-mediated enhancement of NRF2 nuclear accumulation was not dependent on its transcription but possibly depended on the prevention of its degradation. To verify this hypothesis, the protein expression of Keap1, a classic negative regulator of NRF2, was detected using Western blotting. Unexpectedly, treatment with DBZ did not affect Keap1 expression in Ang II-stimulated NRCMs (Figures 9B,C), indicating that the Keap1 was not involved in increasing NRF2 level following DBZ treatment.

According to previous studies, mTOR has a critical role in maintaining oxidative balance and is involved in NRF2 degradation (Mohapatra et al., 2020; Tian et al., 2020). In this study, DBZ significantly inhibited the Ang II-mediated mTOR phosphorylation in NRCMs (Figures 9D,E). To determine whether mTOR was related to the DBZ-mediated inhibition of oxidative stress, _L_-leucine (5 mM) was used to over-activate mTOR. Western blotting results revealed that the _L_-leucine treatment dramatically attenuated DBZ-mediated upregulation of NRF2 and HO-1 and significantly activated mTOR. The DBZ-mediated inhibition of ER stress was almost completely reversed when used with _L_-leucine (Figures 9F–K). In addition, *NRF2* knockdown did not impair the downregulation of mTOR phosphorylation by DBZ in Ang II-stimulated NRCMs (Supplementary Figures S7A,B), suggesting that mTOR acted upstream of NRF2 in Ang II-induced cardiac hypertrophy.

All the findings above suggested that DBZ elevated nuclear NRF2 accumulation by preventing its degradation via the mTOR pathway; however, the exact mechanism of mTOR and NRF2 crosstalk is unknown. Reportedly, the GSK3β/β-TrcP pathway is involved in NRF2 degradation (Chen et al., 2019). Hence, we investigated the influence of DBZ on this pathway. Our results showed that DBZ (10 μm) significantly inhibited the β-TrcP expression and promoted the phosphorylation of GSK3β (Ser9), without significant effects on the expression of total GSK3β in NRCMs (Figures 9L–N). To further clarify whether the inhibition of DBZ on β-TrcP expression was mTOR dependent, the over-activated mTOR was induced by _L_-leucine. Supplementary Figures S7C–F showed that _L_-leucine significantly weakened the regulatory effect of DBZ on GSK3β (Ser 9) and β-TrcP, showing that mTOR is an upstream molecular of β-TrcP. The results suggested that DBZ reduced the NRF2 degradation via the mTOR/β-TrcP/NRF2 pathway rather than the Keap1 pathway.

## Discussion

Currently, treatments for hypertrophic and failing heart diseases are ineffective. So, developing more effective interventions is urgently required. Previous studies have revealed that excessive oxidative and ER stress can perturb the intracellular microenvironment, aggravating cardiac dysfunction (Matsushima et al., 2016; Tian et al., 2021b). ROS production is increased in pressure overloaded cardiac hypertrophy, and the ensuing oxidative damage could, in turn, induce ER stress and apoptosis (Narasimhan et al., 2020). Therefore, compounds with antioxidant effects may be beneficial for preventing the progression of myocardial degeneration. Our study showed for the first time that: 1) DBZ synthesized according to the principles of TCM inhibited oxidative stress, ER stress, and apoptosis in case of TAC-induced cardiac hypertrophy; 2) Although DBZ inhibited autophagy, it did not depend on the regulation of autophagy; 3) DBZ exerted antioxidant effects by enhancing the nuclear accumulation of NRF2, upregulating antioxidant enzymes, and inhibiting ROS-mediated damage; 4) The investigation of the underlying molecular mechanism showed that the above effects of DBZ were strongly involved in the activation of the mTOR/GSK3β (Ser9)/β-TrcP pathway.

In the present subject, TAC and Ang II were used on animal models and cardiomyocytes to induce hypertrophic responses. The results obtained from animal experiments and *in vitro* cellular experiments showed that DBZ significantly reduced the enlargement of cardiomyocytes and expression of hypertrophic markers and attenuated cardiac remodeling. These findings prompted us to speculate that DBZ may act as a novel molecule for the treatment of cardiac hypertrophy. Accumulating evidence suggested that oxidative stress is upregulated in chronic pressure overloaded hearts and strongly correlated with ER stress or autophagy (Camargo et al., 2018; Huo et al., 2021). Therefore, we focused on the antioxidant effects of DBZ and attempted to understand the mechanism underlying these effects. Under stress, cardiomyocytes generate large amounts of ROS, leading to a vicious cycle of stress-induced damage in the heart. However, DBZ could inhibit excess ROS generation mainly through NOX upregulation and scavenging ROS *via* the upregulation of mitochondrial antioxidant enzymes.

As ER stress and autophagy are related to oxidative stress, we investigated whether DBZ can regulate their balance in pathological hypertrophy. We observed that DBZ not only inhibited ER stress but blocked autophagy flow, as evidenced by the presence of stress-related proteins in the ER and autophagy markers. However, whether oxidative stress is the main contributor to ER stress and autophagy remains elusive. Interestingly, when Mito-TEMOP and XO were used to regulate intracellular redox conditions, DBZ attenuated cardiac hypertrophy via downregulation of oxidative stress. Furthermore, we also observed changes in the expression of autophagy proteins upon inhibition of ER stress, indicating that autophagy was reduced when ER stress was restricted. However, an increase in autophagy did not alter the effects of DBZ on cardiac hypertrophy and cell apoptosis. These findings highlighted the importance of inhibiting ROS and ER stress in cardiac hypertrophy, but not autophagy.

NRF2 is a crucial factor in regulating intracellular oxidative stress. Under normal conditions, NRF2 is anchored in the cytoplasm and has low activity; however, it is activated under conditions of ROS surge, increasing the expression of antioxidant enzymes (Chen and Maltagliati, 2017). In our study, DBZ has been shown to increase total cellular NRF2 levels and nuclear NRF2 accumulation. The NRF2 siRNA significantly blocked its antioxidant effects, highlighting that NRF2 was indispensable for the DBZ-mediated inhibition of oxidative stress in cardiac hypertrophy. However, DBZ did not affect NRF2 transcription, showing that it may increase the intracellular NRF2 content by inhibiting its degradation rather than promoting protein synthesis. Thus, further studies were performed to determine whether DBZ downregulated translation of Keap1, a well-known inducer of NRF2 degradation. Our findings showed that Keap1 expression was not regulated by DBZ treatment, indicating that some other mechanisms were involved in NRF2 degradation.

mTOR, an important regulator of cellular stress, mainly promotes oxidative metabolism and mitochondrial biogenesis (Sengupta et al., 2010). Activation of Akt-mTOR signaling enhanced ROS production via NADPH oxidase and mitochondrial dysfunction (Park et al., 2011). In this study, DBZ inhibited stress-induced mTOR phosphorylation, and _L_-leucine nearly eliminated the DBZ-mediated attenuation of oxidative stress and ER stress, indicating that mTOR was pivotal in the DBZ-mediated inhibition of oxidative stress and NRF2 degradation. However, understanding the relationship between mTOR and NRF2 in different cell models is challenging, as they might negatively or positively regulate each other. Based on our results, activation of mTOR with _L_-leucine counteracted DBZ-mediated nuclear NRF2 accumulation, whereas siRNA NRF2 had no effect on DBZ-induced inhibition of mTOR phosphorylation, indicating that mTOR was located upstream of NRF2 in Ang II-stimulated NRCMs.

Although mTOR was an upstream regulatory factor of NRF2, its direct link with NRF2 degradation remains unsolved. Several studies have shown that in addition to Keap1, NRF2 stability is regulated by β-TrcP, an E3 ubiquitin ligase adaptor, which confers specificity in selecting the target substrates for degradation (Rada et al., 2012). GSK3β is a crucial protein involved in NRF2 degradation; it phosphorylates NRF2, facilitating its recognition by β-TrcP for subsequent degradation (Chowdhry et al., 2013). Considering that DBZ inhibited NRF2 degradation, whether it can regulate the GSK3β/β-TrcP pathway to exert its antioxidative effects remains to be investigated. Our findings suggested that DBZ markedly reduced the protein level of β-TrcP and promoted the phosphorylation of GSK3β (a negative regulator of β-TrcP). In addition, mTOR is also located upstream of GSK3β. However, it is still unclear whether DBZ binds to mTOR, which is a limitation of this project. Moreover, the PLN-SERCA2a pathway plays an important role in the pathogenesis of cardiac hypertrophy. The impacts of DBZ on this pathway warrant further investigation because SERCA2a maintains cytoplasmic Ca^2+^ lower during relaxation and determinates sufficient Ca^2+^ release during construction, which is an essential element in protecting cardiac pump function (Asahi et al., 2003). Although our study suggested that the antioxidative effects of DBZ in stress-induced cardiac hypertrophy are mTOR/β-TrCP/NRF2 pathway-dependent, it may not be the only mechanism to benefit cardiac hypertrophy. For instance, the effects of DBZ in regulating the calcineurin-NFAT pathway will be clarified in our future studies (Molkentin, 2004).

More surprisingly, structural analyses of the metabolites showed that enzymes in liver microsomes are responsible for DBZ hydroxylation. The target sites of hydroxylation are on the borneol but not the DSS. These data indicate that DSS is the main active metabolite of DBZ, and it may be effective as a prodrug for the clinical treatment of diseases (Liu et al., 2010). Moreover, for nongenetic dilated cardiomyopathy (DCM), resulting mainly from myocarditis, long-term exposure to alcohol, drugs, or metabolic and endocrine disturbances are reportedly closely linked with transforming growth factor (TGF)-beta, RAAS, and angiotensin II in DCM development. DBZ may be a great potential therapeutic drug for its anti-inflammatory and anti-stress properties (Reichart et al., 2019).

In conclusion, for the first time, we showed that DBZ robustly protects against cardiac hypertrophy in rats after TAC surgery and in cardiomyocytes treated with Ang II, which is mostly due to the antioxidant and anti-ER stress properties of DBZ, via the mTOR/GSK3β(Ser9)/β-TrcP/NRF2 pathway. These findings imply that the novel compound, DBZ, may be considered a potential drug candidate for treating cardiac hypertrophy associated with excessive stress, such as heart failure.

## Data Availability

The original contributions presented in the study are included in the article/Supplementary Material, further inquiries can be directed to the corresponding authors.
