# Supplementary material for: A Novel Compound, Tanshinol Borneol Ester, Ameliorates Pressure Overload-Induced Cardiac Hypertrophy by Inhibiting Oxidative Stress via the mTOR/β-TrCP/NRF2 Pathway
Source: Front Pharmacol. 2022 Feb 3;13:830763. doi: 10.3389/fphar.2022.830763 (PMC8850779; doi:10.3389/fphar.2022.830763)

Supplementary Information for

**A novel compound, tanshinol borneol ester, ameliorates pressure overload-induced cardiac hypertrophy by inhibiting oxidative stress via the mTOR/β-TrCP/NRF2 pathway**

**Supplementary methods**

**Terminal deoxynucleotidyl transferase-mediated dUTP-biotin nick end labeling (TUNEL) staining.**

The paraffin sections of myocardial tissues were dewaxed, treated with 3% hydrogen peroxide solution for 10 min, washed with phosphate-buffered saline (PBS) for 3 times, and subjected to TUNEL staining according to the instructions of the TUNEL kit (Beyotime, Shanghai, China). After staining, sections were added with anti-fluorescence quenching liquid for sealing, and the number of apoptotic myocardial cells was observed and calculated.

**Wheat Germ Agglutinin (WGA) Staining**

The heart was cut in half by forming a transverse slice. The base specimen was fixed in 10% formalin buffer, embedded in paraffin, and cut into 5-μm thick sections. The cross-sectional area of cardiomyocytes was measured in images captured in sections stained with 5 μM WGA (Thermo, MA, United States).

**F-actin (Phalloidin) staining**

The cardiomyocytes were washed with PBS before fixation with 4% formaldehyde in PBS at room temperature for 15 min, and then rinsed with PBS for three times (5 min each). The fixed cells were incubated with Alexa Fluor 488 Phalloidin (1:20, Cell Signaling Technology, Danvers, MA, USA) for 15 min at room temperature. After rinsing with PBS, nuclei were counterstained by 4’,6-diamidino-2-phenylindole (DAPI; 10 ng/ml, ThermoFisher Scientific, Waltham, MA, USA). The fluorescent images were captured using a confocal microscope.

**Mitochondrial antioxidant enzymes detection**

The primary mitochondrial antioxidant enzymes were quantified using commercially available kits purchased from Beyotime Institute of Biotechnology (Shanghai, China). Heart tissues were ground into homogenates and centrifuged at 3000 × *g* for 15 min, collecting the supernatants, then the superoxide dismutase-1 (SOD-1), glutathione peroxidase (GPx), and malondialdehyde (MDA) contents was quantified according to the manufacturer’s instructions.

**Supplementary Table**

**Table** Details of primers used for validation through RT-PCR

| **Gene name** | **Primer** |
| --- | --- |
| GAPDH | F: AGGTCGGTGAACGGATTTG |
|  | R: TGTAGACCATGTAGTTGAGGTCA |
| Nppa | F: GGCTTCTTCCTCTTCCTGGC |
|  | R: TGTGTTGGACACCGCACTGT |
| Myh7 | F: CTGGCACCGTGGACTACA |
|  | R: TTGGCAAACAGATTACTTAGGA |
| Procollagen I | F: GTGCTCCTGGTATTGCTGGT |
|  | R: GGCTCCTCGTTTTCCTTCTT |
| Procollagen III | F: TGGAAAACCAGGAGAACCAG |
|  | R: CAGACCAGGAGGACCAGAAG |
| Nrf2 | F: TCTTGGAGTAAGTCGAGAAGTGT |
|  | R: GTTGAAACTGAGCGAAAAAGGC |

**Supplementary Figures**

**
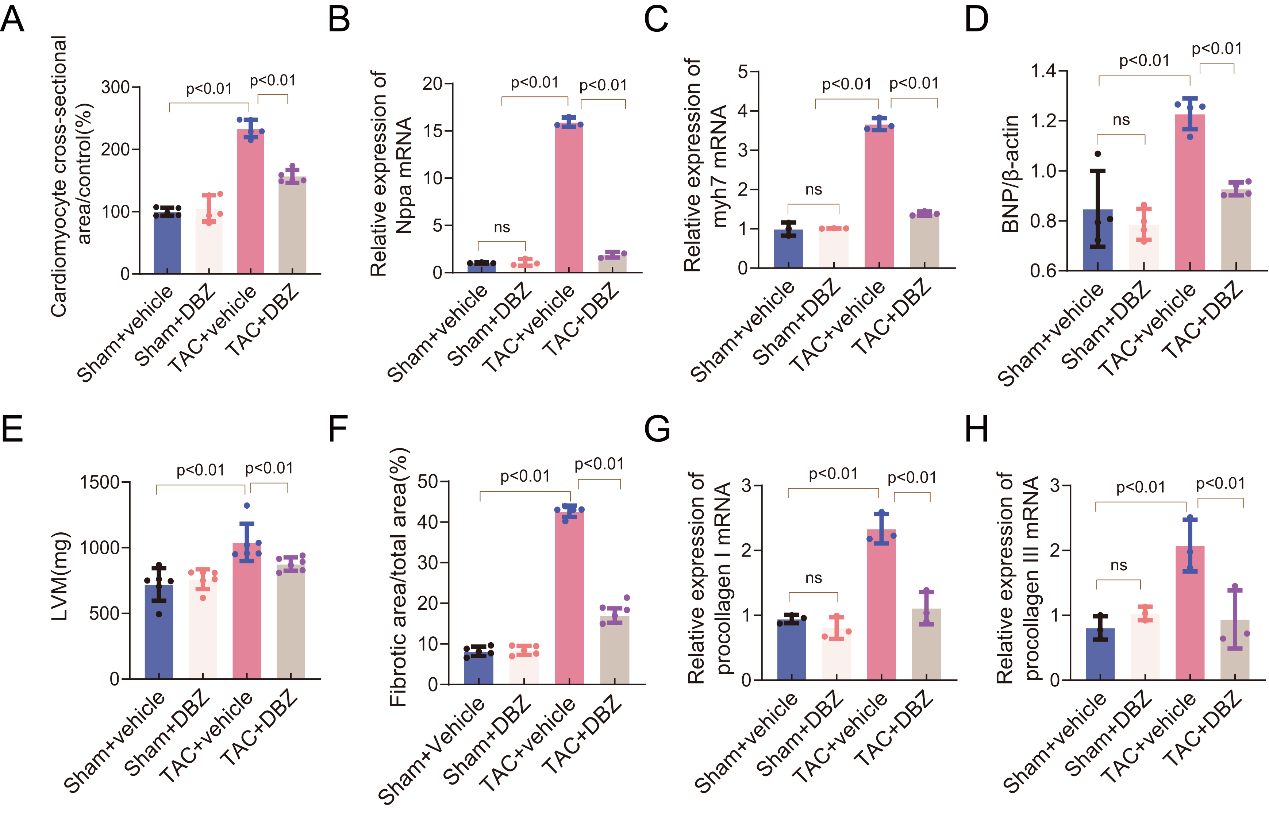
**

**Fig. S1 DBZ improves cardiac function and attenuates cardiac remodeling.** (A) Quantification of cross-section areas of cardiomyocytes. n = 5. (B-C) Real-time PCR quantification of relative mRNA expression level of Nppa and myh7. n = 4. (D) Quantification of BNP expression. n = 4. (E) Quantification of left ventricular mass (LVM). n = 6. (F) Quantification of fibrotic area in myocardium sections. n = 5. (G-H) Real-time PCR quantification of relative mRNA expression level of procollagen I and procollagen III. n = 3. Results are expressed as means ± SD. Statistical analyses were performed by one-way ANOVA followed by Bonferroni’s *post hoc* test.


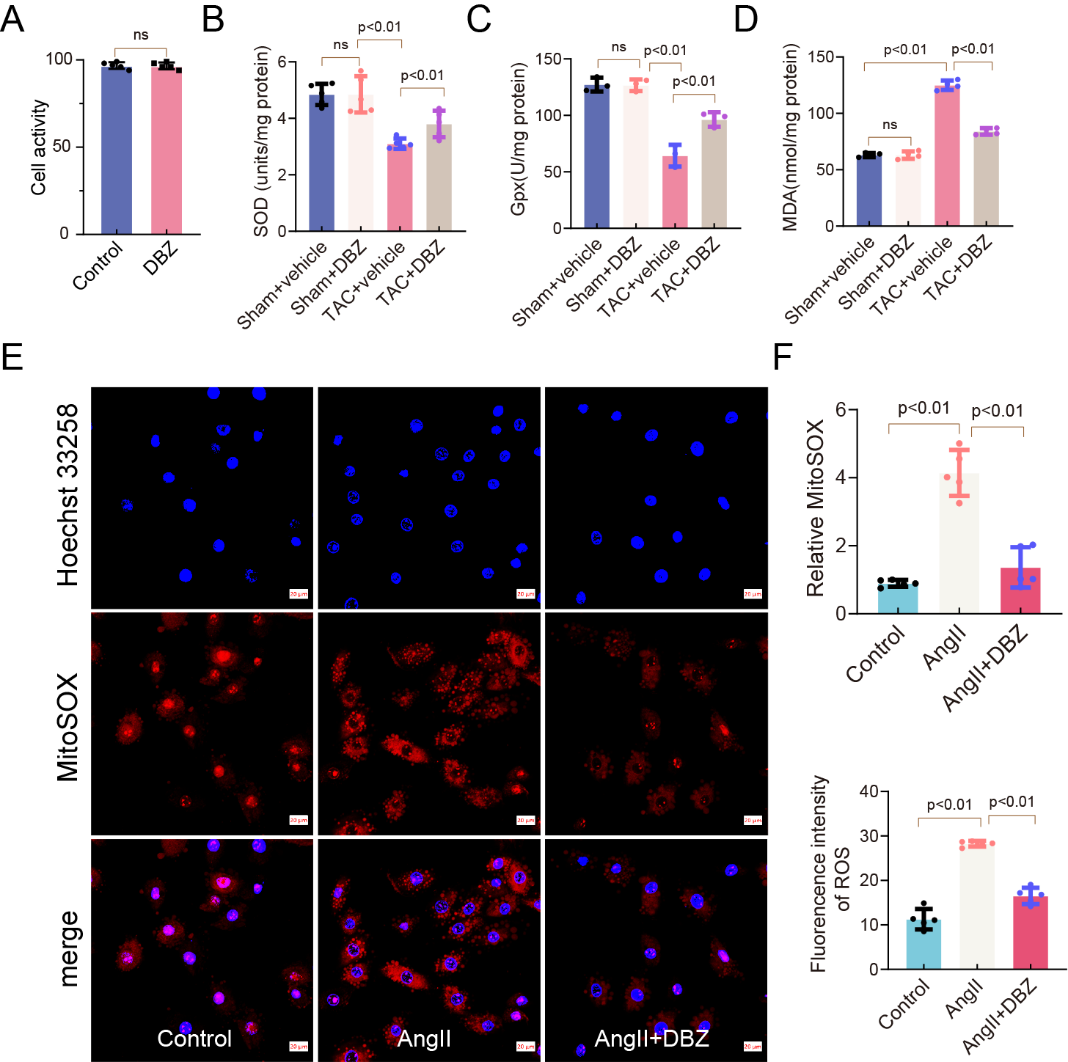


**Fig. S2 DBZ attenuated the oxidative stress.** (A) Cell activity were detected using CCK-8 kit. n = 3-5. (B-D) SOD activity, Gpx activity and MDA levels. n = 3-5. (E-F) Fluorescence intensity of MitoSOX and quantification of fluorescence intensity of ROS. n = 5. Results are expressed as means ± SD. Statistical analyses were performed by one-way ANOVA followed by Bonferroni’s *post hoc* test.


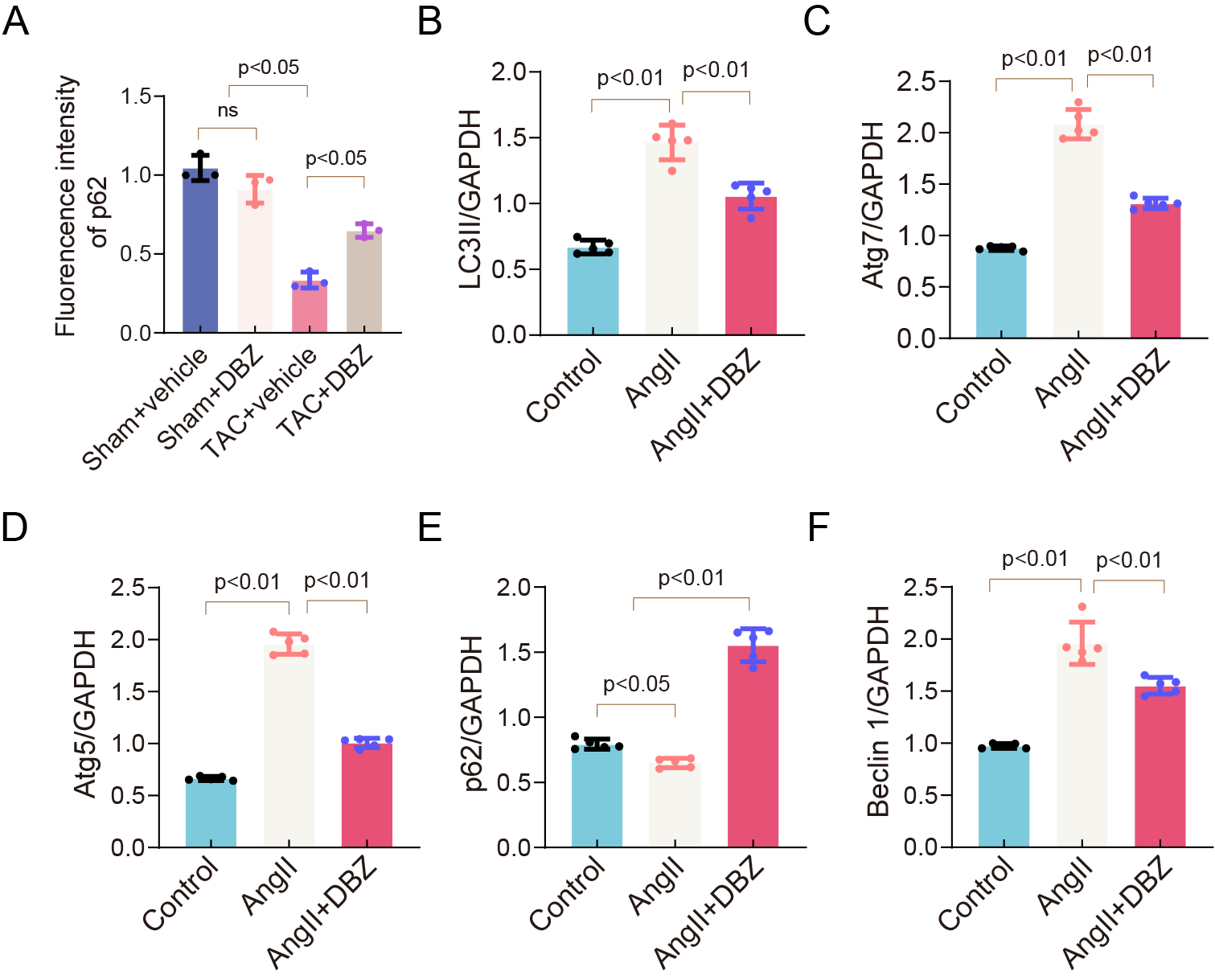


**Fig. S3 DBZ inhibited autophagy.** (A) Statistics of mean fluorescent intensity of p62. (B-F) Quantification of LC3, Atg7, Atg5, p62 and Beclin1 expression. n = 5. Results are expressed as means ± SD. Statistical analyses were performed by one-way ANOVA followed by Bonferroni’s *post hoc* test.


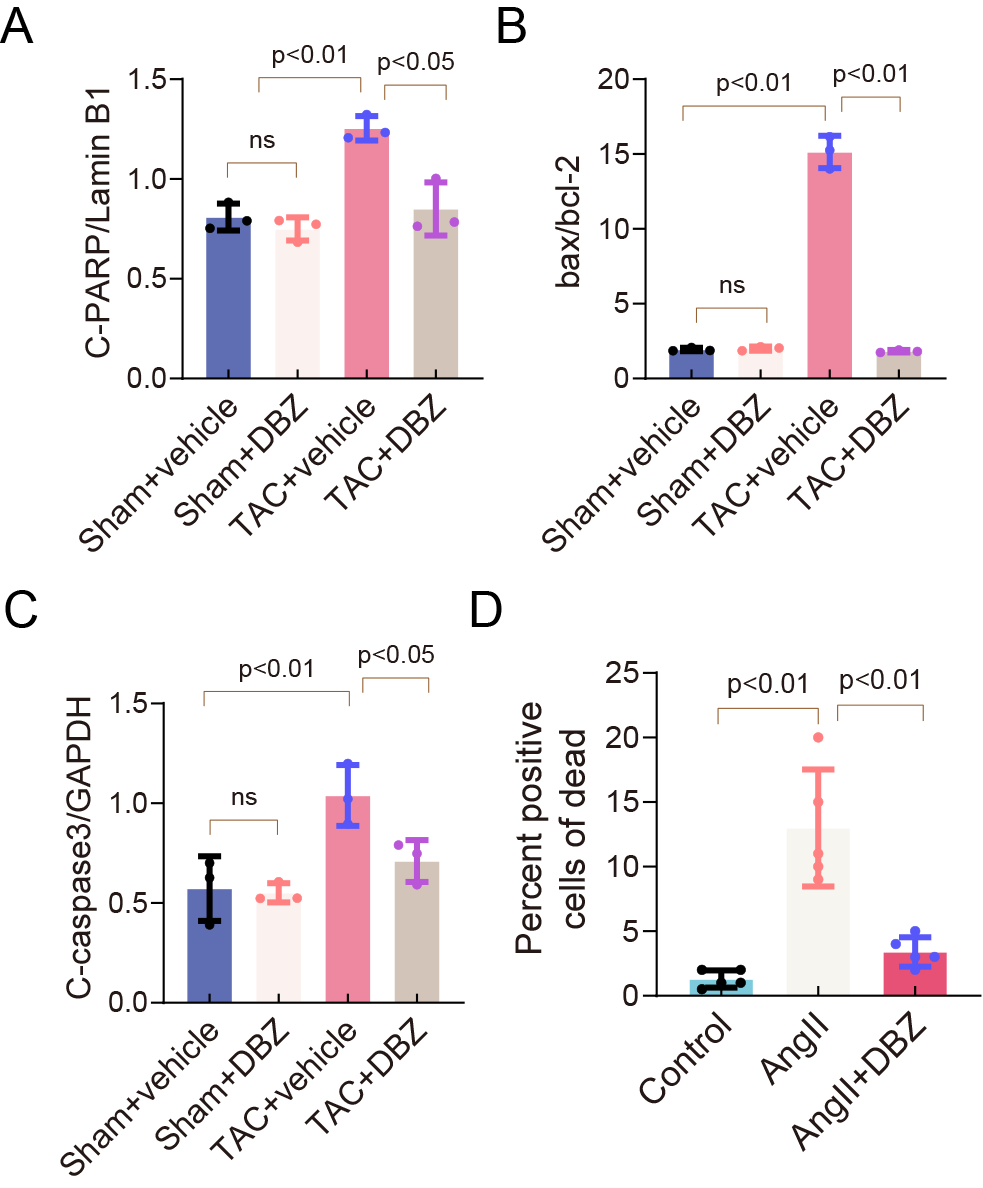


**Fig. S4 DBZ ameliorated apoptosis. (A-C)** Quantification of C-PARP, bax/bcl-2, and C-caspase 3 expression. n = 3. (D) Quantification of dead cells. n = 5. Data are expressed as means ± SD. Statistical analyses were performed by one-way ANOVA followed by Bonferroni’s *post hoc* test.


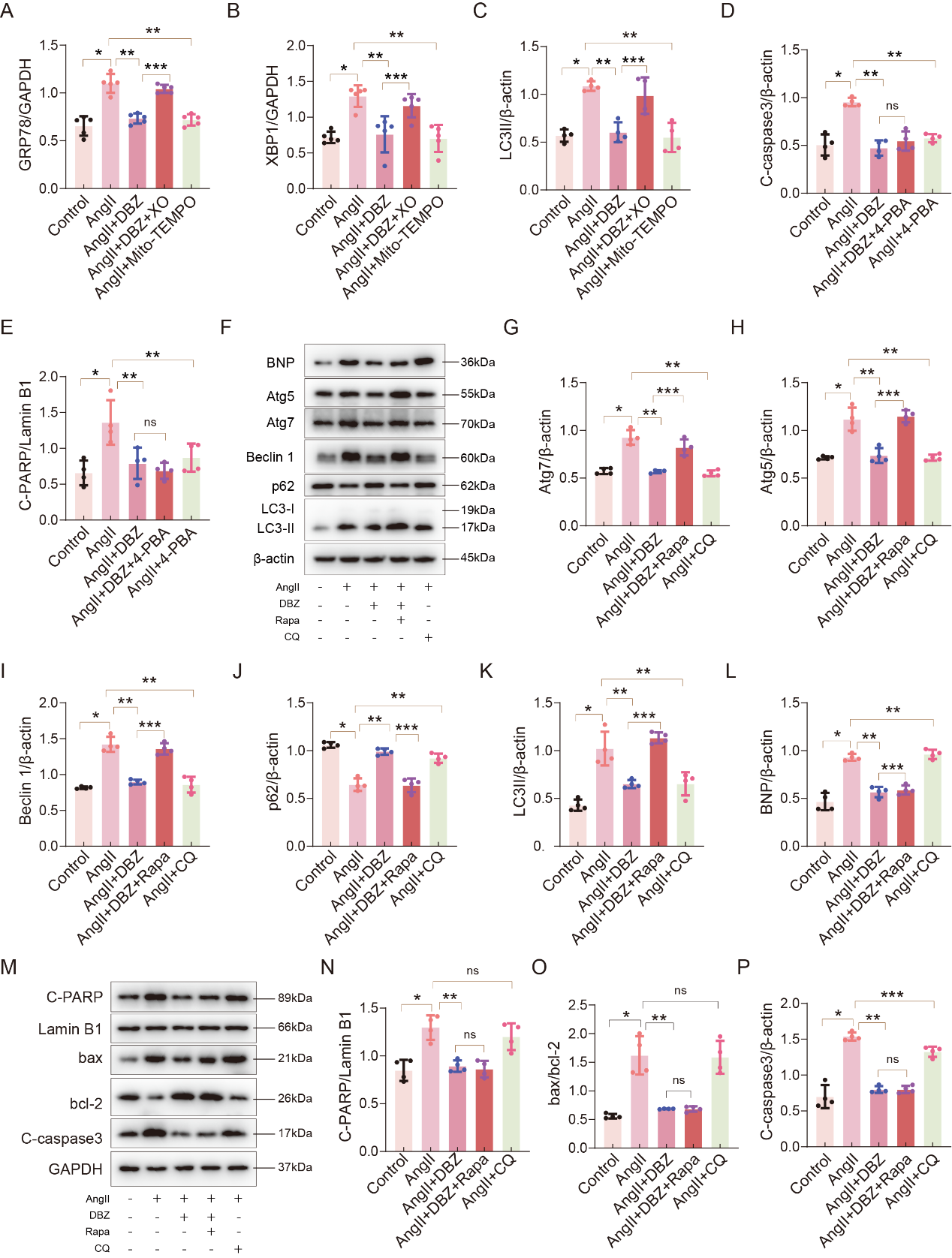


**Fig. S5 ROS contributed to ER stress, autophagy and apoptosis.** (A-E) Quantification of GRP78, XBP1, LC3, C-caspase 3, and C-PARP expression. n = 4-5. (F-L) Representative Western blotting assay and quantification of BNP, Atg5, Atg7, Beclin1, p62, and LC3 expression. n = 4. (M-P) Representative Western blotting assay and quantification of C-PARP, bax, bcl-2, and C-caspase 3 expression. n = 4. *p < 0.05 compared with the Control group, **p < 0.05 compared with the Ang II group, ***p < 0.05 compared with the Ang II + DBZ group. Data are expressed as means ± SD. Statistical analyses were performed by one-way ANOVA followed by Bonferroni’s *post hoc* test.


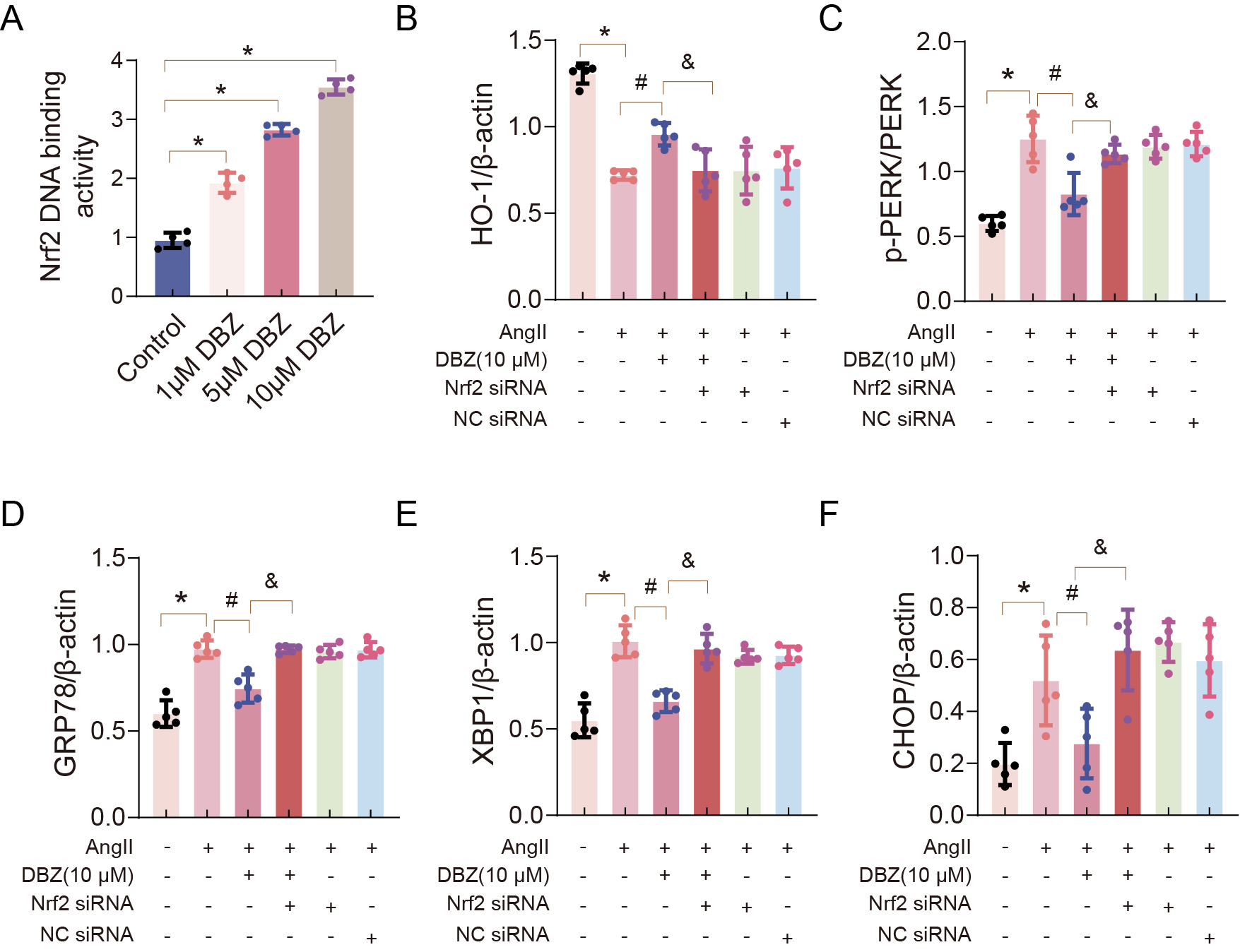


**Fig. S6 DBZ increased the nuclear accumulation of Nrf2.** (A) Detection of the Nrf2 binding activity. n = 4. (B-F) Quantification of HO-1, p-PERK, GRP78, XBP1, and CHOP expression. n = 5. *p < 0.05 compared with the Control group, **p < 0.05 compared with the Ang II group, ***p < 0.05 compared with the Ang II + DBZ group. Data are expressed as means ± SD. Statistical analyses were performed by one-way ANOVA followed by Bonferroni’s *post hoc* test.

**
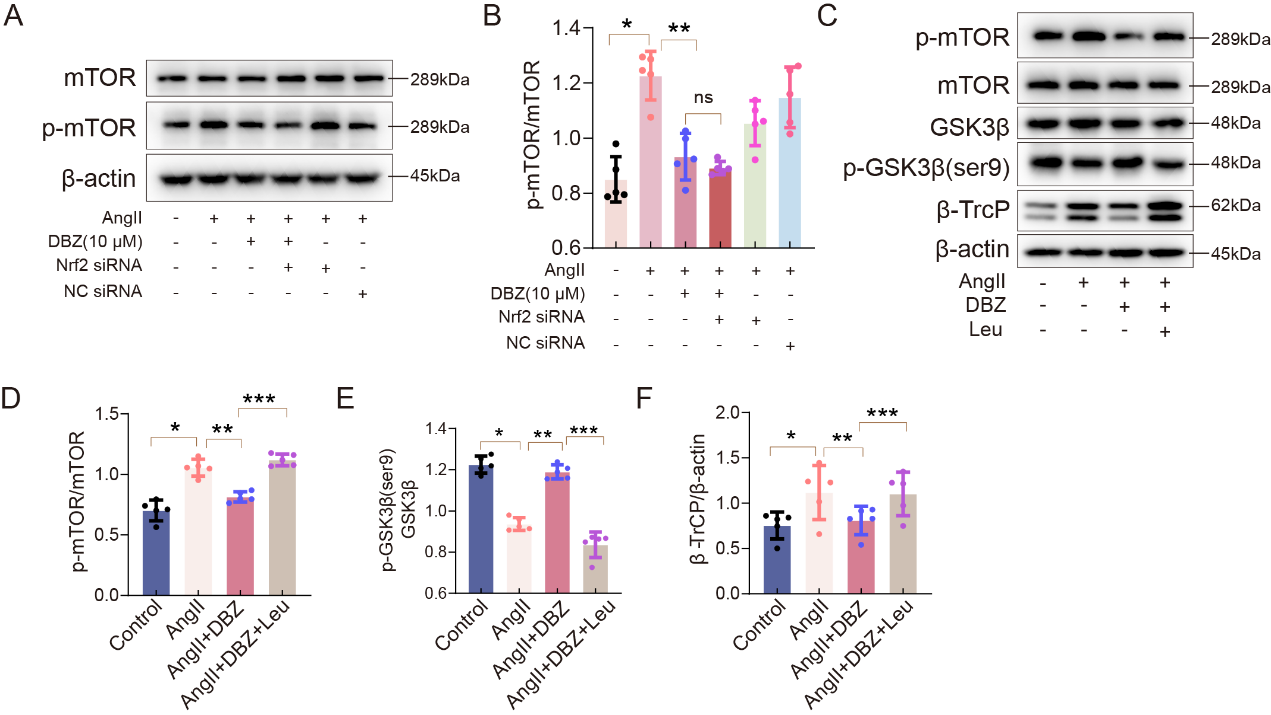
**

**Fig. S7 DBZ inhibited the degradation of Nrf2.** (A-B) Representative Western blotting assay and quantification of p-mTOR and mTOR expression. n = 5. (C-F) Representative Western blotting assay and quantification of p-mTOR , mTOR, GSK3β, p-GSK3β(ser9) and β-TrcP expression. n = 5. *p < 0.05 compared with the Control group, **p < 0.05 compared with the Ang II group, ***p < 0.05 compared with the Ang II + DBZ group. Data are expressed as means ± SD. Statistical analyses were performed by one-way ANOVA followed by Bonferroni’s *post hoc* test.

**Original images of Western blotting**


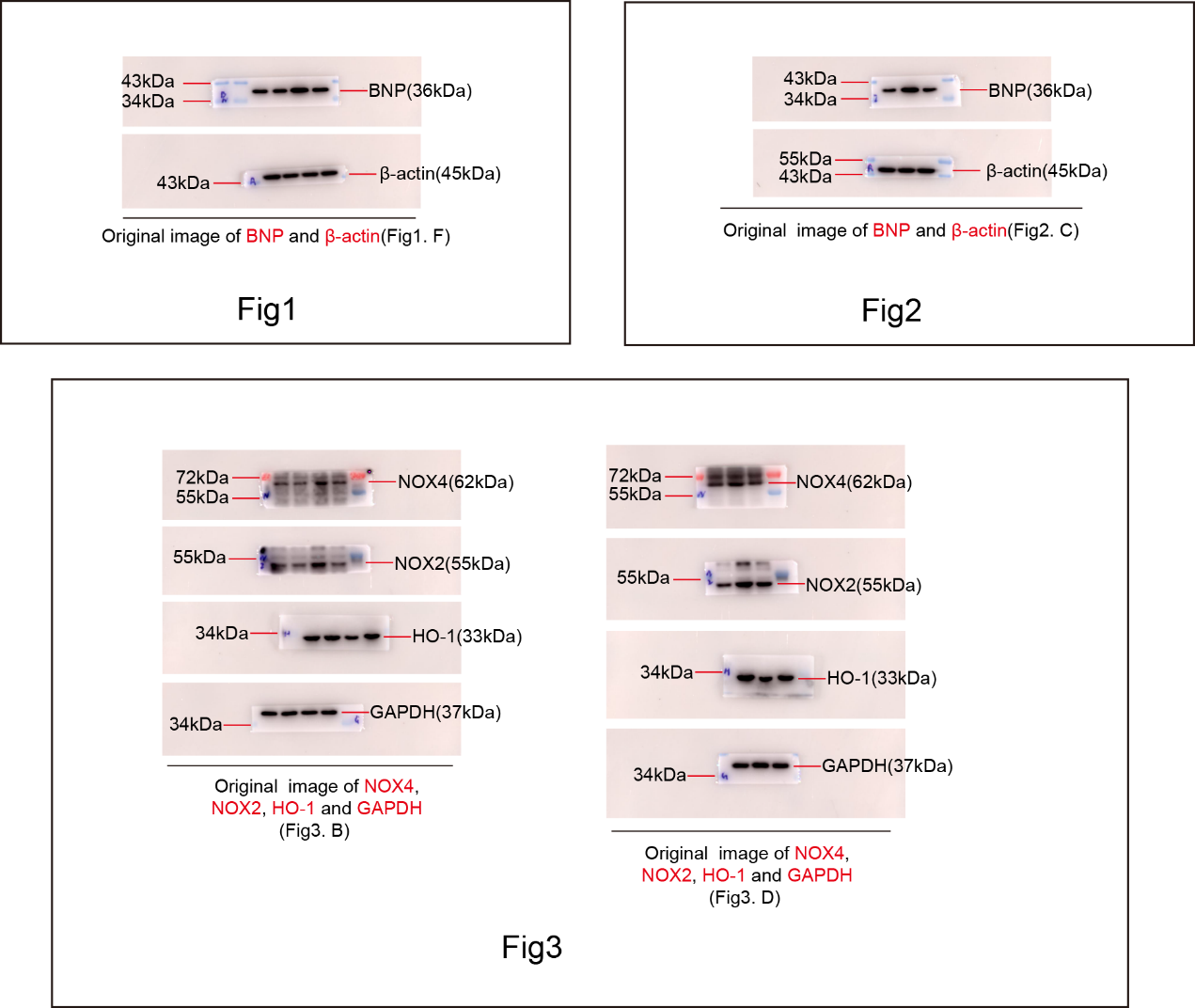


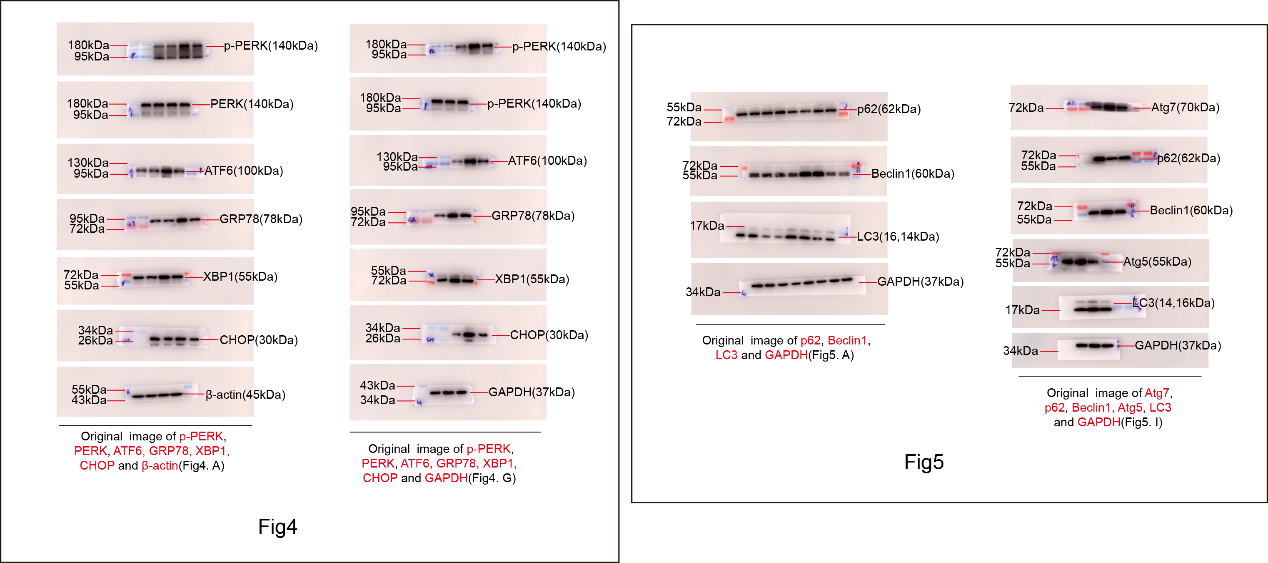


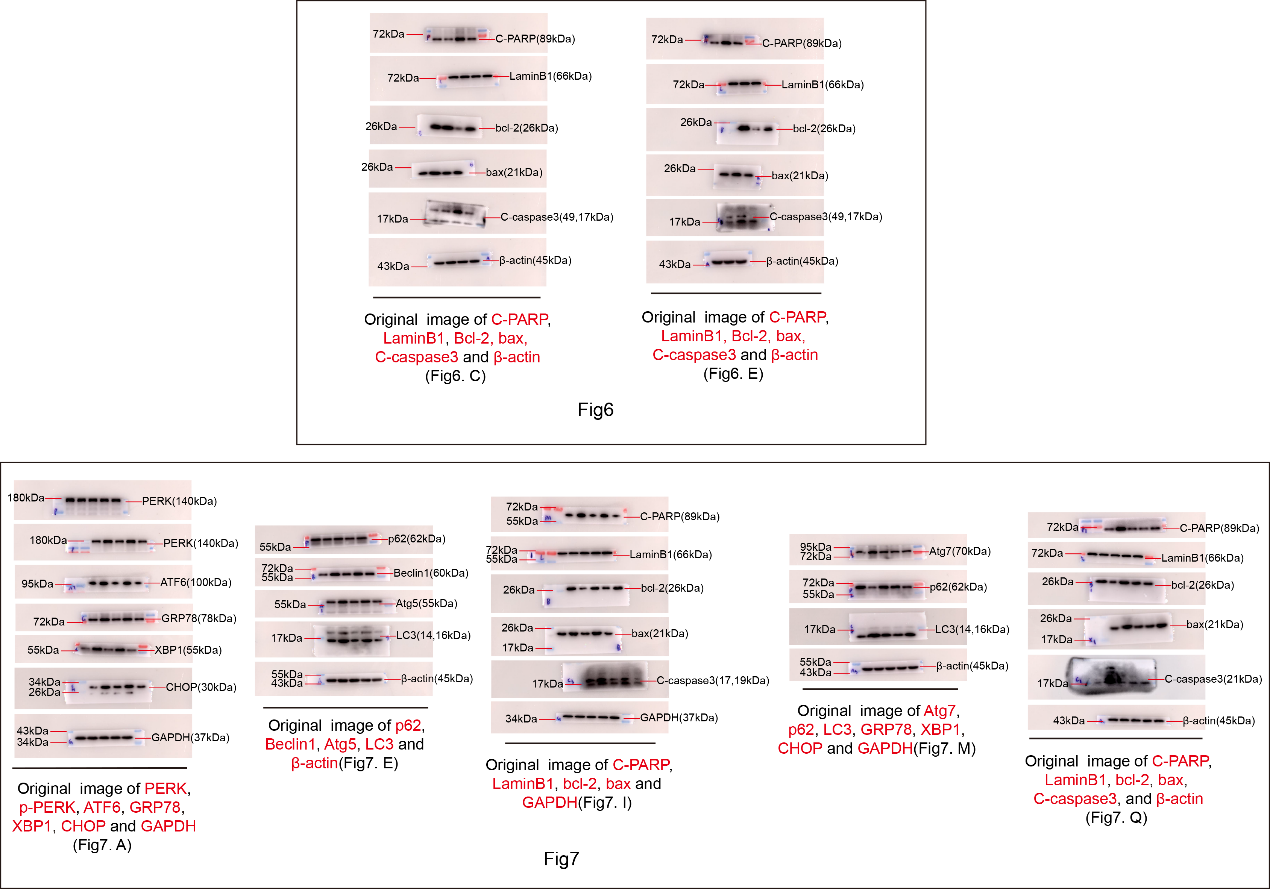


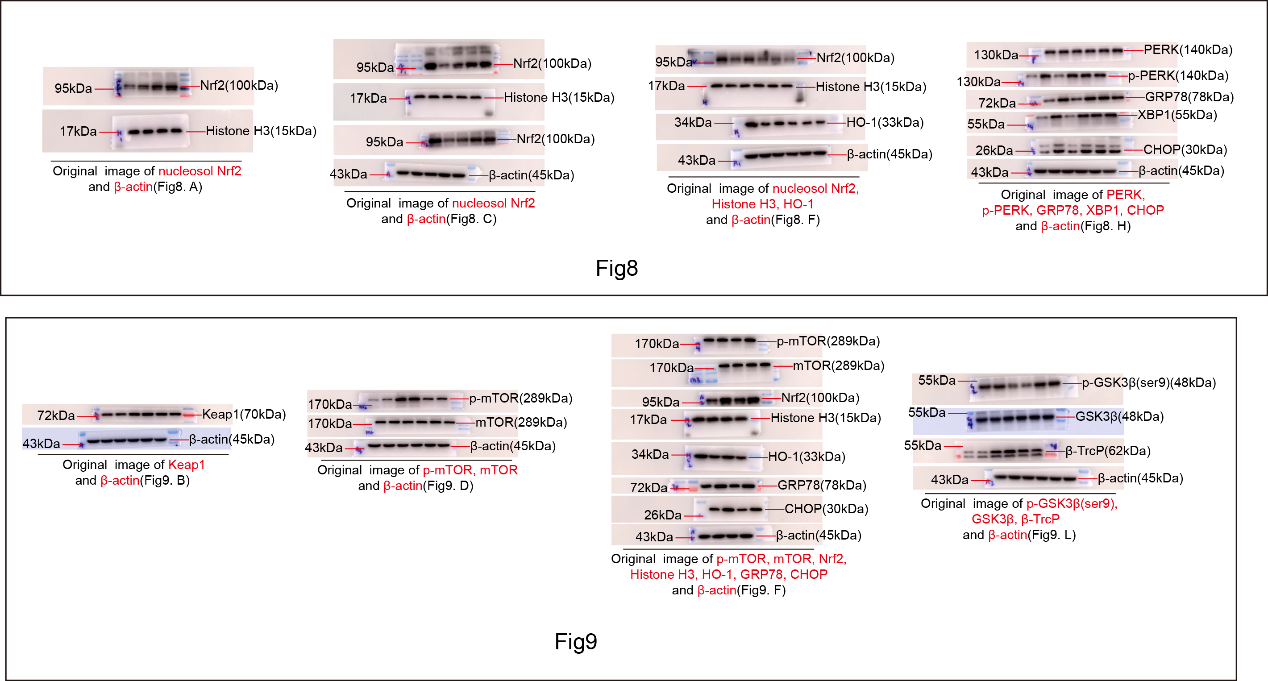


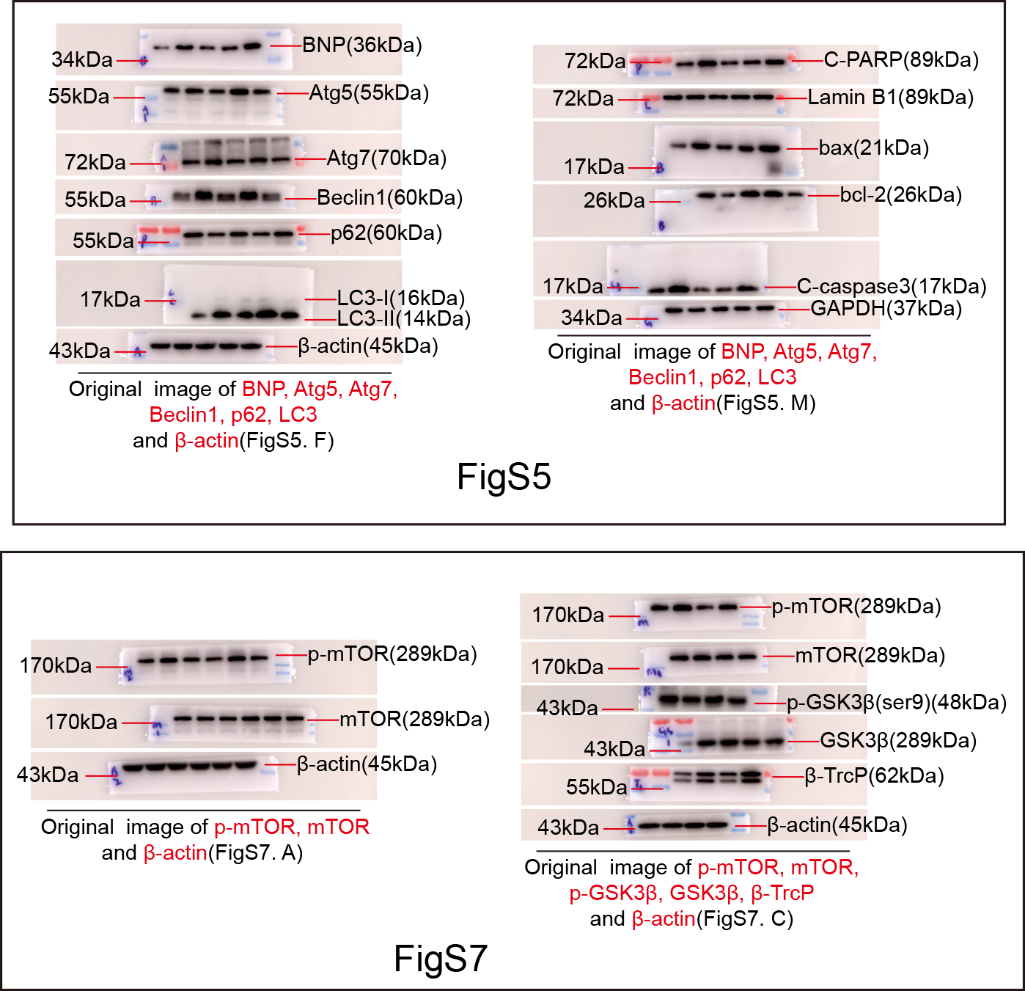

Supplement: Supplementary file 1 [file DataSheet1.docx]
